# Metal(II) Complexes of the Fluoroquinolone Fleroxacin: Synthesis, Characterization and Biological Profile

**DOI:** 10.3390/pharmaceutics14050898

**Published:** 2022-04-20

**Authors:** Alexandra Kostelidou, Franc Perdih, Jakob Kljun, Foteini Dimou, Stavros Kalogiannis, Iztok Turel, George Psomas

**Affiliations:** 1 Department of General and Inorganic Chemistry, Faculty of Chemistry, Aristotle University of Thessaloniki, GR-54124 Thessaloniki, Greece; ale-25-kost@hotmail.com; 2 Faculty of Chemistry and Chemical Technology, University of Ljubljana, Večna pot 113, 1000 Ljubljana, Slovenia; franc.perdih@fkkt.uni-lj.si (F.P.); jakob.kljun@fkkt.uni-lj.si (J.K.); 3 Department of Nutritional Sciences and Dietetics, International Hellenic University, GR-57400 Thessaloniki, Greece; dimoufot@yahoo.gr (F.D.); kalogian@ihu.gr (S.K.)

**Keywords:** fleroxacin, metal(II) complexes, structure, interaction with DNA, affinity for albumins, antimicrobial activity

## Abstract

A series of complexes of divalent transition metals (Cu(II), Mn(II), Zn(II), Co(II) and Ni(II)) with the quinolone antibacterial agent fleroxacin, in the absence or presence of an *α*-diimine such as 2,2′-bipyridine, 1,10-phenanthroline or 2,2′-bipyridylamine, were prepared and characterized. The complexes were characterized by various physicochemical and spectroscopic techniques and by single-crystal X-ray crystallography. The in vitro antibacterial activity of the complexes was studied against the bacterial strains *Staphylococcus aureus*, *Bacillus subtilis* and *Xanthomonas campestris* and was higher than that of free quinolone. The affinity of the complexes for bovine and human serum albumin was studied by fluorescence emission spectroscopy and the determined binding constants showed tight and reversible binding to the albumins. The interaction of the complexes with calf-thymus DNA was studied by various techniques, which showed that intercalation was the most plausible mode of interaction.

## 1. Introduction

Fluoroquinolones are a class of synthetic antimicrobial agents that have been increasingly used in treatment since 1979 and the synthesis of norfloxacin [1,2]. At that time, it was discovered that the incorporation of a fluorine atom into the quinolone structure significantly increased the antimicrobial activity of quinolones [3]. Nowadays, despite numerous negative side-effects [4], fluoroquinolones are among the most important antimicrobial agents [5] because they have broad-spectrum antimicrobial activity against Gram(+) and Gram(−) microorganisms and are used for the treatment of community-acquired pneumonia, respiratory tract infections and urinary tract infections [6]. It is also worth noting that studies have demonstrated the potential action of fluoroquinolones for the treatment of SARS-CoV-2-associated pneumonia, and drugs from this family were also recommended in the treatment of community-acquired pneumonia in COVID-19 patients [7,8].

The main biological targets of fluoroquinolones are gyrase and topoisomerase IV, the enzymes mainly involved in the DNA-unwinding during replication [9,10]. A plethora of transition metal complexes bearing quinolones as ligands have been prepared and characterized, as reported in the literature [11,12]. Most of these complexes have shown similar or improved biological profiles compared to free quinolones [12,13]. However, it is also well known that the bioavailability of fluoroquinolone drugs may be reduced due to interactions with the metal ions found in antacids and drug formulations [14,15].

**Figure 1:**
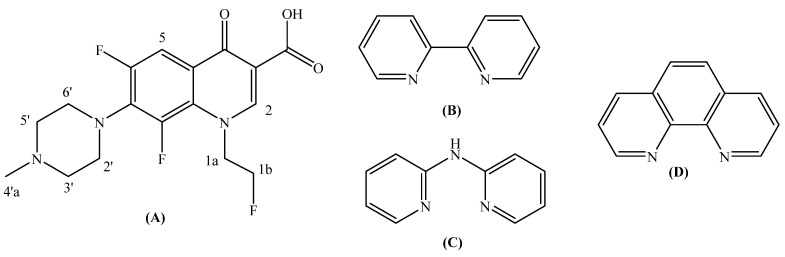
(**A**) The syntax formula and H-atom numbering of fleroxacin (Hflrx). (**B**–**D**) The *N,N*′-donors used as co-ligands: (**B**) 2,2′-bipyridine (bipy). (**C**) 2,2′-bipyridylamine (bipyam) and (**D**) 1,10-phenanthroline (phen).

Fleroxacin (Hflrx = 6,8-difluoro-1-(2–fluoroethyl)-7-(4-methylpiperazin-1-yl)-4-oxoquinoline-3-carboxylic acid, Figure 1A) is a second-generation trifluorinated fluoroquinolone with a broad spectrum of activity [16,17]. Its main mechanism of action is the inhibition of the DNA-supercoiling activity of DNA gyrase [18]. Its major advantage over other fluoroquinolones is its long half-life, which allows dosing once daily [19], although it appears to have adverse side effects [20]. Despite its extensive use as a fluoroquinolone, there are few reports on the synthesis, characterization and biological activity of metal complexes with fleroxacin, including a Zn(II) (i.e., [Zn(flrx)(phen)(H_2_O)](NO_3_), phen = 1,10-phenanthroline) [21], a Ga(III) (i.e., [Ga(flrx)_3_]) [22], a ^99m^Tc [23] and two Cu(II) complexes (i.e., the cationic complex [Cu(flrx)(phen)(H_2_O)](CH_3_COO)·9.8H_2_O [24] and the neutral complex [Cu(flrx)(bipy)Cl]·4H_2_O, bipy = 2,2′-bipyridine [25]).

Continuing the research project on the characterization and biological evaluation of metal complexes with quinolones as ligands [12,26,27,28,29,30,31,32,33,34], we selected the quinolone fleroxacin and prepared a series of its coordination compounds with divalent ions of transition metals. More specifically, a series of copper(II), manganese(II), zinc(II), cobalt(II) and nickel(II) complexes were synthesized with fleroxacin as a ligand in the absence or presence of the *N,N*′-donor co-ligands 2,2′-bipyridine (bipy), 1,10-phenanthroline (phen) or 2,2′-bipyridylamine (bipyam) (Figure 1). The complexes were characterized by elemental analysis, room-temperature (RT) magnetic measurements, IR, UV–vis and ^1^H NMR spectroscopies, mass spectrometry and single-crystal X-ray crystallography.

The in vitro antimicrobial activity of the compounds was evaluated by determining the minimum inhibitory concentration (MIC) against the Gram-positive microorganisms *Staphylococcus aureus* ATCC 6538 (*S. aureus*) and *Bacillus subtilis* ATCC 6633 (*B. subtilis*) and the Gram-negative microorganism *Xanthomonas campestris* ATCC 1395 (*X. campestris*). The affinity of the compounds for bovine serum albumin (BSA) and human serum albumin (HSA) was studied by fluorescence emission spectroscopy, and the corresponding binding constants were determined. Furthermore, the interaction of the compounds with calf-thymus DNA (CT DNA) was investigated to determine the possible mode of interaction by the following methods: (i) UV–vis spectroscopy (where the DNA-binding constants of the complexes (K_b_) were also determined), (ii) DNA-viscosity measurements, (iii) cyclic voltammetry and (iv) competitive DNA-binding studies with the classic intercalator ethidium bromide (EB) performed by fluorescence emission spectroscopy.

## 2. Materials and Methods

### 2.1. Materials—Instrumentation—Physical Measurements

All chemicals (i.e., CuCl_2_·2H_2_O, MnCl_2_∙4H_2_O, CoCl_2_·6H_2_O, NiCl_2_·6H_2_O, ZnCl_2_, KOH, fleroxacin, bipy, phen, bipyam, trisodium citrate, NaCl, BSA, HSA, CT DNA, EB) and solvents were reagent-grade and were used as purchased from commercial sources, without further purification.

Infrared spectra were recorded in the range 400–4000 cm^−1^ on a Nicolet FT–IR 6700 spectrometer as the samples were prepared as KBr pellets (abbreviations used: (s) for strong, (m) for medium, (br) for broad, (w) for weak). The electronic (ultraviolet–visible, UV–vis) spectra of the compounds (as nujol mulls and in DMSO solutions (C = 10^–5^ – 5 × 10^–3^ M)) were recorded on a Hitachi U-2001 dual-beam spectrophotometer. The C, H and N elemental analyses were performed on a PerkinElmer 240B elemental microanalyzer. The molar conductivity measurements of the complexes (1 mM DMSO solution) were performed with a Crison Basic 30 conductometer. The magnetic measurements were performed at RT on a magnetic susceptibility balance of Johnson Matthey Chemicals Limited by the Faraday method. The fluorescence emission spectra of the compounds were recorded in solution on a Hitachi F-7000 fluorescence spectrophotometer. The viscosity experiments were performed at 100 rpm on an ALPHA L Fungilab rotational viscometer using an 18 mL LCP spindle. ^1^H NMR spectra were recorded on an Agilent 500/54 (500 MHz for ^1^H) spectrometer using DMSO-*d*_6_ as solvent.

A Thermo TSQ Quantum Access MAX triple quadrupole mass spectrometer (Thermo Fisher; Waltham, MA, USA) was used (positive ESI–MS(+) or negative ESI–MS(–) electrospray ionization). The mass spectrometer runs in a full-scan technique detecting the parent mass of each compound. For operation in MS/MS mode, the values of collision energy and tube lens were optimized for each compound separately. The capillary temperature was set to 350 °C and the vaporizer temperature of the ESI probe to 70 °C.

A stock solution of CT DNA was prepared by dissolving into a buffer (containing 150 mM NaCl and 15 mM trisodium citrate at pH 7.0) and was stirred at 4 °C for 3 days. Afterwards, it was kept at 4 °C for no longer than a week. This stock DNA solution gave a ratio of UV absorbance at 260 and 280 nm (A_260_/A_280_) of 1.85–1.90, which indicated that DNA was sufficiently free of protein contamination [35]. The concentration per nucleotide of this stock DNA solution was determined by the UV absorbance at 260 nm using ε = 6600 M^−1^ cm^−1^ [36].

### 2.2. Synthesis of the Complexes

#### 2.2.1. General Synthetic Procedure for Complexes [M(flrx)_2_(O-Donor)_x_] (x = 0 or 2) (Complexes **1**–**5**)

For the synthesis of complexes **1**–**5**, a methanolic solution containing fleroxacin (0.5 mmol, 185 mg) and KOH (0.5 mmol, 0.5 mL 1 M) was stirred for 60 min. Afterwards, the solution was added into a methanolic solution of the corresponding salt MCl_2_ (0.25 mmol) at RT. The reaction mixture was stirred for 1 h and left to slowly evaporate. After a few days, (micro)crystalline products were collected.

**[Cu(flrx)_2_], 1:** For the synthesis of complex **1**, CuCl_2_·2H_2_O (43 mg, 0.25 mmol) was used as MCl_2_. Light-blue product of **1** (110 mg, 55%) was collected after three days. *Anal*. calc. for [Cu(flrx)_2_] (C_34_H_34_CuF_6_N_6_O_6_) (MW = 800.22). C: 51.03, H: 4.29, N: 10.51%; found: C: 51.30, H: 4.15, N: 10.39%. ESI–MS(+), *m*/*z*: found, 799.0; calc. for MW, 800.2.IR (KBr disk), *ν*_max_ (cm^−1^): *ν*(C=O)_pyr_, 1619 (s); *ν*_asym_(COO), 1589 (s); *ν*_sym_(COO), 1371 (s); Δ*ν*(COO) = *ν*_asym_(COO) –*ν*_sym_(COO) = 218. UV–vis: as nujol mull, λ (in nm): 665, 395; in DMSO, λ (in nm) (ε, in M^–1^ cm^–1^): 680 (55), 392 (shoulder (sh)) (340), 335 (5900), 320 (3500), 293 (10,500). μ_eff_ at RT = 1.86 BM. The complex is soluble in DMSO (Λ_M_ = 3 mho·cm^2^·mol^−1^, in 1 mM DMSO solution) and partially soluble in methanol and DMF.

**[Mn(flrx)_2_(H_2_O)_2_], 2:** For the synthesis of complex **2**, ΜnCl_2_·4H_2_O (49 mg, 0.25 mmol) was used as the MCl_2_. Yellow product of **2** (115 mg, 55%) was collected after a month. *Anal*. calc. for [Mn(flrx)_2_(H_2_O)_2_] (C_34_H_48_F_6_MnN_6_O_8_) (MW = 827.64). C: 49.34, H: 4.63, N: 10.15%; found: C: 49.30, H: 4.85, N: 10.29%. ESI–MS(+), *m*/*z*: found, 878.19; calc. for MW + 1 + H_2_O + MeOH, 878.70. IR (KBr disk), *ν*_max_ (cm^−1^): *ν*(C=O)_pyr_, 1619 (s); *ν*_asym_(COO), 1573 (s); *ν*_sym_(COO), 1372 (s); Δ*v*(COO) = 201. UV–vis: as nujol mull, λ (in nm): 405 (sh); in DMSO, λ (in nm) (ε, in M^–1^ cm^–1^): 385(sh) (190), 333 (4500), 288 (8500). μ_eff_ at RT = 6.05 BM. The complex is soluble in methanol, DMF and DMSO (Λ_M_ = 3 mho·cm^2^·mol^−1^, in 1 mM DMSO solution).

**[Zn(flrx)_2_(MeOH)_2_]·2MeOH, 3·2MeOH:** For the synthesis of complex **3**, ZnCl_2_ (34 mg, 0.25 mmol) was used as the MCl_2_. Off-white crystals of **3** (120 mg, 55%) suitable for X-ray crystallography were collected after three weeks. *Anal*. calc. for [Zn(flrx)_2_(MeOH)_2_] (C_36_H_42_F_6_N_6_O_8_Zn) (MW = 866.14). C: 49.92, H: 4.89, N: 9.70%; found: C: 50.15, H: 4.95, N: 9.47%. ESI–MS(+), *m*/*z*: found, 899.12; calc. for MW+1+MeOH, 899.14. IR (KBr disk), *ν*_max_ (cm^–1^): *ν*(C=O)_pyr_, 1618 (s); *ν*_asym_(COO), 1580 (s); *ν*_sym_(COO), 1373 (s); Δ*ν*(COO) = 207. UV–vis: as nujol mull, λ (in nm): 401 (sh); in DMSO, λ (in nm) (ε, in M^–1^ cm^–1^): 387(sh) (250), 333 (4900), 290 (10,000). ^1^H NMR spectrum in DMSO-*d*_6_, δ (in ppm): 8.85 (2H, H^2^–flrx), 7.79 (2H, H^5^–flrx), 4.88–4.78 (8H, H^1b^– and H^1a^–flrx), 4.59 (6H, H–MeOH), 3.40 (8H, H^2′,6′^–flrx), 2.44 (8H, H^3′,5′^–flrx), 2.19 (6H, H^4′a^–flrx). The complex is soluble in DMSO (Λ_M_ = 7 mho·cm^2^·mol^−1^, in 1 mM DMSO solution) and partially soluble in DMF and methanol.

**[Co(flrx)_2_(MeOH)_2_], 4:** For the synthesis of complex **4**, CoCl_2_·6H_2_O (59 mg, 0.25 mmol) was used as the MCl_2_. Orange microcrystalline product of **4** (125 mg, 60%) was collected after a month. *Anal*. calc. for [Co(flrx)_2_(MeOH)_2_] (C_36_H_42_CoF_3_N_6_O_8_) (MW = 859.69). C: 50.30, H: 4.92, N: 9.78%; found: C: 50.45, H: 4.75, N: 9.54%. ESI–MS(+), *m*/*z*: found, 886.24. IR (KBr disk), *ν*_max_ (cm^–1^): *ν*(C=O)_pyr_, 1615 (s); *ν*_asym_(COO), 1578 (s); *ν*_sym_(COO), 1372 (s); Δ*ν*(COO) = 206. UV–vis: as nujol mull, λ (in nm): 634, 525, 425 (sh); in DMSO, λ (in nm) (ε, in M^−1^ cm^−1^): 630 (sh) (15), 540 (25), 435 (sh) (45), 385(sh) (170), 331, (3200), 288 (6200). μ_eff_ at RT = 4.15 BM. The complex is soluble in methanol, DMF and DMSO (Λ_M_ = 5 mho·cm^2^·mol^–1^, in 1 mM DMSO solution) and partially soluble in CH_3_CN and CH_2_Cl_2_.

**[Ni(flrx)_2_(MeOH)_2_], 5:** For the synthesis of complex **5**, NiCl_2_·6H_2_O (59 mg, 0.25 mmol) was used as the MCl_2_. Green product of **5** (120 mg, 55%) was collected after two months. *Anal*. calc. for [Ni(flrx)_2_(MeOH)_2_] (C_36_H_42_F_6_N_6_NiO_8_) (MW = 859.47). C: 50.31, H: 4.93, N: 9.78%; found: C: 50.18, H: 4.78, N: 9.69%. ESI–MS(+), *m*/*z*: found, 889.81; calc. for MW + MeOH, 891.47.IR (KBr disk), *ν*_max_ (cm^–1^): *ν*(C=O)_pyr_, 1619 (s); *ν*_asym_(COO), 1578 (s); *ν*_sym_(COO), 1372 (s); Δv(COO) = 207. UV–vis: as nujol mull, λ (in nm): 975, 630, 401(sh); in DMSO, λ (in nm) (ε, in M^−1^ cm^−1^): 995 (10), 615 (20), 395 (sh) (60), 335 (2700), 289 (7100); 10Dq = 10050 cm^−1^, B = 761 cm^−1^, 10Dq/B = 13.2. μ_eff_ at RT = 2.90 BM. The complex is soluble in DMF and DMSO (Λ_M_ = 5 mho·cm^2^·mol^−1^, in 1 mM DMSO solution) and partially soluble in methanol.

#### 2.2.2. General Synthetic Procedure for Complexes [Cu(flrx)(*N*,*N*′-Donor)Cl] (*N*,*N*′-Donor = bipy, bipyam, phen), (Complexes **6**–**8**)

The complexes of the formula [Cu(flrx)(*N*,*N*′-donor)Cl] were prepared by the addition of a methanolic solution of fleroxacin (0.25 mmol, 92 mg), which was deprotonated by KOH (0.25 mmol, 0.25 mL 1 M) after 1 h stirring into a methanolic solution (5 mL) of CuCl_2_·2H_2_O (43 mg,0.25 mmol) at RT, simultaneously with a methanolic solution (5 mL) of the corresponding *N,N*′-donor (0.25 mmol). The reaction mixture was stirred for additional 15 min. After a few days, the (micro)crystalline product was formed and collected with filtration.

**[Cu(flrx)(bipy)Cl], 6:** For the synthesis of complex **6**, bipy (39 mg, 0.25 mmol) was used as the *N,N*′-donor. Blue crystals of **6** (75 mg, 50%) suitable for X-ray crystallography were collected after two weeks. *Anal*. calc. for [Cu(flrx)(bipy)Cl] (C_27_H_25_ClCuF_3_N_5_O_3_) (MW = 614.91). C: 52.74, H: 4.10, N: 11.39%; found: C: 52.50, H: 3.99, N: 11.30%. IR (KBr disk), *ν*_max_ (cm^−1^): *ν*(C=O)_pyr_, 1620 (s); *ν*_asym_(COO), 1589 (s); *ν*_sym_(COO), 1363 (s); Δ*ν*(COO) = 226; ρ(C–H)_bipy_, 778 (m). UV–vis: as nujol mull, λ (in nm): 665, 402 (sh); in DMSO, λ (in nm) (ε, in M^−1^ cm^−1^): 645 (95), 389 (sh) (220), 333 (2800), 315 (3500), 294 (7900). μ_eff_ at RT = 1.80 BM. The complex is soluble in methanol, DMF and DMSO (Λ_M_ = 5 mho·cm^2^·mol^−1^, in 1 mM DMSO solution) and partially soluble in CH_3_CN and CH_2_Cl_2_.

**[Cu(flrx)(bipyam)Cl], 7:** For the synthesis of complex **7**, bipyam (43 mg, 0.25 mmol) was used as the *N,N*′-donor. Blue–green crystals of **7** (85 mg, 55%) suitable for X-ray crystallography were collected after a month. *Anal*. calc. for [Cu(flrx)(bipyam)Cl] (C_27_H_26_ClCuF_3_N_6_O_3_) (MW = 638.54). C: 50.79, H: 4.10, N: 13.16%; found: C: 51.05, H: 4.02, N: 12.94%. IR (KBr disk), *ν*_max_ (cm^−1^): *ν*(C=O)_pyr_, 1621 (s); *ν*_asym_(COO), 1585 (s); *ν*_sym_(COO), 1373 (s); Δ*ν*(COO) = 212; ρ(C–H)_bipyam_, 781 (m). UV–vis: as nujol mull, λ (in nm): 655, 405; in DMSO, λ (in nm) (ε, in M^−1^ cm^−1^): 660 (80), 390(sh) (200), 345 (sh) (2100), 319 (5100), 295 (7500). μ_eff_ at RT = 1.78 BM. The complex is soluble in MeOH, CH_3_CN, DMF and DMSO (Λ_M_ = 7 mho·cm^2^·mol^–1^, in 1 mM DMSO solution).

**[Cu(flrx)(phen)Cl], 8:** For the synthesis of complex **8**, phen (45 mg, 0.25 mmol) was used as the *N,N*′-donor. Blue microcrystalline product of **8** (95 mg, 60%) was collected after a week. *Anal*. calc. for [Cu(flrx)(phen)Cl] (C_29_H_25_ClCuF_3_N_5_O_3_) (MW = 647.54). C: 53.79, H: 3.89, N: 10.81%; found: C: 53.55, H: 3.75, N: 11.04%. ESI–MS(+), *m/z*: found, 649.9; calc. for MW+1, 648.5. IR (KBr disk), *ν*_max_ (cm^−1^): *ν*(C=O)_pyr_, 1620 (s); *ν*_asym_(COO), 1587 (s); *ν*_sym_(COO), 1371 (s); Δ*ν*(COO) = 216; ρ(C–H)_phen_, 724 (m). UV–vis: as nujol mull, λ (in nm): 670, 401; in DMSO, λ (in nm) (ε, in M^−1^ cm^−1^): 675 (65), 390 (sh) (250), 358 (sh) (1500), 329 (2700), 294 (8100). μ_eff_ at RT = 1.80 BM. The complex is soluble in MeOH, DMF and DMSO (Λ_M_ = 4 mho·cm^2^·mol^−1^, in 1 mM DMSO solution) and partially soluble in CH_3_CN.

#### 2.2.3. General Synthetic Procedure for Complexes [M(flrx)_2_(*N,N*′-Donor)_2_] (M(II) = Mn(II), Zn(II), Co(II), Ni(II), and *N,N*′-Donor = bipy, bipyam, phen) (Complexes **9**–**19**)

The complexes of the formula [M(flrx)_2_(*N,N*′-donor)_2_] were prepared by the addition of a methanolic solution of fleroxacin (0.5 mmol, 185 mg), which was deprotonated by KOH (0.5 mmol, 0.5 mL 1 M) after 1 h stirring into a methanolic solution (5 mL) of MCl_2_ (0.25 mmol) at RT, simultaneously with a methanolic solution (5 mL) of the corresponding *N,N*′-donor (0.25 mmol). The reaction mixture was stirred for additional 30 min. After a few days, the (micro)crystalline product was formed and collected with filtration.

**[Mn(flrx)_2_(bipy)], 9:** For the synthesis of complex **9**, ΜnCl_2_·4H_2_O (49 mg, 0.25 mmol) was used as the MCl_2_ and bipy (39 mg, 0.25 mmol) as the *N,N*′-donor. Beige crystals of **9** (120 mg, 50%) suitable for X-ray crystallography were collected after two months. *Anal*. calc. for [Mn(flrx)_2_(bipy)] (C_44_H_42_F_6_MnN_8_O_6_) (MW = 947.79). C: 55.76, H: 4.47, N: 11.88%; found: C: 55.50, H: 4.29, N: 11.69%. ESI–MS(+), *m*/*z*: found, 974.86. IR (KBr disk), *ν*_max_ (cm^−1^): *ν*(C=O)_pyr_, 1618(s); *ν*_asym_(COO), 1579 (s); *ν*_sym_(COO), 1372 (s); Δ*ν*(COO) = 207; ρ(C–H)_bipy_, 760(m). UV–vis: as nujol mull, λ (in nm): 405 (sh); in DMSO, λ (in nm) (ε, in M^−1^ cm^−1^): 405 (sh) (190), 333 (3400), 286 (6800). μ_eff_ at RT = 5.95 BM. The complex is soluble in MeOH, DMF and DMSO (Λ_M_ = 11 mho·cm^2^·mol^–1^, in 1 mM DMSO solution).

**[Mn(flrx)_2_(bipyam)], 10:** For the synthesis of complex **10**, ΜnCl_2_·4H_2_O (49 mg, 0.25 mmol) was used as the MCl_2_ and bipyam (43 mg, 0.25 mmol) as the *N,N*′-donor. Yellowish microcrystalline product of **10** (120 mg, 50%) was collected after a month. *Anal*. calc. for [Mn(flrx)_2_(bipyam)] (C_44_H_43_F_6_MnN_9_O_6_) (MW = 962.81). C: 54.90, H: 4.50, N: 13.09%; found: C: 54.70, H: 4.59, N: 12.89%. ESI–MS(+), *m*/*z*: found, 992.01; calc. for MW + MeOH, 994.8. IR (KBr disk), *ν*_max_ (cm^−1^): *ν*(C=O)_pyr_, 1616 (s); *ν*_asym_(COO), 1574 (s); *ν*_sym_(COO), 1372 (s); Δ*ν*(COO) = 202; ρ(C–H)_bipyam_, 762 (m). UV–vis: as nujol mull, λ (in nm): 402 (sh); in DMSO, λ (in nm) (ε, in M^−1^ cm^−1^): 390 (sh) (220), 316 (6500), 287 (6500). μ_eff_ at RT = 5.90 BM. The complex is soluble in DMF and DMSO (Λ_M_ = 10 mho·cm^2^·mol^–1^, in 1 mM DMSO solution) and partially soluble in methanol.

**[Mn(flrx)_2_(phen)], 11:** For the synthesis of complex **11**, ΜnCl_2_·4H_2_O (49 mg, 0.25 mmol) was used as the MCl_2_ and phen (45 mg, 0.25 mmol) as the *N,N*′-donor. Yellow product of **11** (135 mg, 55%) was collected after two months. *Anal*. calc. for [Mn(flrx)_2_(phen)] (C_46_H_42_F_6_MnN_8_O_6_) (MW = 971.82). C: 56.85, H: 4.36, N: 11.54%; found: C: 56.70, H: 4.22, N: 11.39%. ESI–MS(+), *m*/*z*: found, 1025.96. IR (KBr disk), *ν*_max_ (cm^−1^): *ν*(C=O)_pyr_, 1616 (s); *ν*_asym_(COO), 1578 (s); *ν*_sym_(COO), 1372 (s); Δ*ν*(COO) = 206; ρ(C–H)_phen_, 731 (m). UV–vis: as nujol mull, λ (in nm): 405 (sh); in DMSO, λ (in nm) (ε, in M^−1^ cm^−1^): 395 (sh) (200), 357 (sh) (1900), 332 (3500), 285 (9500). μ_eff_ at RT = 5.92 BM. The complex is soluble in MeOH, DMF and DMSO (Λ_M_ = 4 mho·cm^2^·mol^−1^, in 1 mM DMSO solution).

**[Zn(flrx)_2_(bipy)], 12:** For the synthesis of complex **12**, ZnCl_2_ (34 mg, 0.25 mmol) was used as the MCl_2_ and bipy (39 mg, 0.25 mmol) as the *N,N*′-donor. Yellowish product of **12** (130 mg, 55%) suitable for X-ray crystallography were collected after a month. *Anal*. calc. for [Zn(flrx)_2_(bipy)] (C_44_H_42_F_6_N_8_O_6_Zn) (MW = 958.24). C: 55.15, H: 4.42, N: 11.69%; found: C: 55.30, H: 4.25, N: 11.49%. ESI–MS(+), *m*/*z*: found, 966.01. IR (KBr disk), *ν*_max_ (cm^−1^): *ν*(C=O)_pyr_, 1619 (s); *ν*_asym_(COO), 1578 (s); *ν*_sym_(COO), 1372 (s); Δ*ν*(COO) = 206; ρ(C–H)_bipy_, 769 (m). UV–vis: as nujol mull, λ (in nm): 405 (sh); in DMSO, λ (in nm) (ε, in M^−1^ cm^−1^): 388(sh) (480), 332 (3500), 288 (7500). ^1^H NMR spectrum in DMSO-*d*_6_, δ (in ppm): 8.69 (4H, H^2^–flrx and H^3,3′^–bipy), 8.42 (2H, H^6,6′^–bipy), 7.99 (4H, H^5^–flrx and H^5,5′^–bipy), 7.50 (2H, H^4,4′^–bipy), 4.82– 4.73 (8H, H^1b^– and H^1a^–flrx), 3.25 (8H, H^2′,6′^–flrx), 2.40 (8H, H^3′,5′^–flrx), 2.19 (6H, H^4′a^–flrx). The complex is soluble in MeOH, DMF and DMSO (Λ_M_ = 9 mho·cm^2^·mol^−1^, in 1 mM DMSO solution).

**[Zn(flrx)_2_(bipyam)], 13:** For the synthesis of complex **13**, ZnCl_2_ (34 mg, 0.25 mmol) was used as the MCl_2_ and bipyam (43 mg, 0.25 mmol) as the *N,N*′-donor. Light-yellow product of **13** (130 mg, 50%) was collected after six weeks. *Anal*. calc. for [Zn(flrx)_2_(bipyam)] (C_44_H_43_F_6_N_9_O_6_Zn) (MW = 973.25). C: 54.30, H: 4.45, N: 12.95%; found: C: 54.50, H: 4.39, N: 12.79%. ESI–MS(+), *m*/*z*: found, 955.16. IR (KBr disk), *ν*_max_ (cm^−1^): *ν*(C=O)_pyr_, 1616 (s); *ν*_asym_(COO), 1578 (s); *ν*_sym_(COO), 1372 (s); Δ*ν*(COO) = 206; ρ(C–H)_bipyam_, 775 (m). UV–vis: as nujol mull, λ (in nm): 406 (sh); in DMSO, λ (in nm) (ε, in M^−1^ cm^−1^): 392 (sh) (280), 323 (4100), 2+0 (6500). ^1^H NMR spectrum in DMSO-*d*_6_, δ (in ppm): 9.68 (1H, H^1^–bipyam), 8.73 (2H, H^2^–flrx), 8.20 (2H, H^3,3′^–bipyam), 7.69 (2H, H^5^–flrx), 7.61–7.67 (4H, H^5,5′,6,6′^–bipyam), 6.68 (2H, H^4,4′^–bipyam), 4.85–4.76 (8H, H^1b^– and H^1a^–flrx), 3.37 (8H, H^2′,6′^–flrx), 2.42 (8H, H^3′,5′^–flrx), 2.21 (6H, H^4′a^–flrx). The complex is soluble in DMF and DMSO (Λ_M_ = 8 mho·cm^2^·mol^−1^, in 1 mM DMSO solution) and partially soluble in methanol.

**[Zn(flrx)_2_(phen)], 14:** For the synthesis of complex **14**, ZnCl_2_ (34 mg, 0.25 mmol) was used as the MCl_2_ and phen (45 mg, 0.25 mmol) as the *N,N*′-donor. Off-yellow product of **14** (125 mg, 50%) was collected after two months. *Anal*. calc. for [Zn(flrx)_2_(phen)] (C_46_H_42_F_6_N_8_O_6_Zn) (MW = 982.26). C: 56.25, H: 4.31, N: 11.41%; found: C: 56.30, H: 4.05, N: 11.59%. ESI–MS(–), *m*/*z*: found, 978.92. IR (KBr disk), *ν*_max_ (cm^–1^): *ν*(C=O)_pyr_, 1620 (s); *ν*_asym_(COO), 1579 (s); *ν*_sym_(COO), 1370 (s); Δ*ν*(COO) = 209; ρ(C–H)_phen_, 730 (m). UV–vis: as nujol mull, λ (in nm): 403 (sh); in DMSO, λ (in nm) (ε, in M^−1^ cm^−1^): 385 (sh) (190), 331 (2800), 288 (6400). ^1^H NMR spectrum in DMSO-*d*_6_, δ (in ppm): 9.06 (2H, H^2,9^–phen), 8.84 (2H, H^4,7^–phen), 8.70 (2H, H^2^–flrx), 8.23 (2H, H^5,6^–phen), 8.06 (2H, H^3,8^–phen), 7.32 (2H, H^5^–flrx), 4.81–4.73 (8H, H^1b^– and H^1a^–flrx), 3.22 (8H, H^2′,6′^–flrx), 2.38 (8H, H^3′,5′^–flrx), 2.18 (6H, H^4′a^–flrx). The complex is soluble in MeOH, DMF and DMSO (Λ_M_ = 9 mho·cm^2^·mol^−1^, in 1 mM DMSO solution) and partially soluble in CH_3_CN and CH_2_Cl_2_.

**[Co(flrx)_2_(bipy)], 15:** For the synthesis of complex **15**, CoCl_2_·6H_2_O (59 mg, 0.25 mmol) was used as the MCl_2_ and bipy (39 mg, 0.25 mmol) as the *N,N*′-donor. Orange product of **15** (120 mg, 50%) was collected after ten weeks. *Anal*. calc. for [Co(flrx)_2_(bipy)] (C_44_H_42_CoF_6_N_8_O_6_) (MW = 951.79). C: 55.53, H: 4.45, N: 11.77%; found: C: 55.30, H: 4.25, N: 11.59%. ESI–MS(+), *m*/*z*: found, 946.89. IR (KBr disk), *ν*_max_ (cm^–1^): *ν*(C=O)_pyr_, 1621 (s); *ν*_asym_(COO), 1578 (s); *ν*_sym_(COO), 1372 (s); Δ*ν*(COO) = 207; ρ(C–H)_bipy_, 773 (m). UV–vis: as nujol mull, λ (in nm): 615 (sh), 522, 415 (sh); in DMSO, λ (in nm) (ε, in M^−1^ cm^−1^): 620 (sh) (15), 535 (25), 435 (sh) (55), 385 (sh) (130), 332 (3500), 287 (5900). μ_eff_ at RT = 3.98 BM. The complex is soluble in MeOH, DMF and DMSO (Λ_M_ = 6 mho·cm^2^·mol^−1^, in 1 mM DMSO solution) and partially soluble in CH_3_CN and CH_2_Cl_2_.

**[Co(flrx)_2_(bipyam)], 16:** For the synthesis of complex **16**, CoCl_2_·6H_2_O (59 mg, 0.25 mmol) was used as the MCl_2_ and bipyam (43 mg, 0.25 mmol) as the *N,N*′-donor. Orange product of **16** (135 mg, 55%) was collected after two months. *Anal*. calc. for [Co(flrx)_2_(bipyam)] (C_44_H_43_CoF_6_N_9_O_6_) (MW = 966.81). C: 54.66, H: 4.48, N: 13.04%; found: C: 54.40, H: 4.65, N: 12.89%. ESI–MS(+), *m*/*z*: found, 981.55. IR (KBr disk), *ν*_max_ (cm^−1^): *ν*(C=O)_pyr_, 1620 (s); *ν*_asym_(COO), 1579 (s); *ν*_sym_(COO), 1372 (s); Δ*ν*(COO) = 207; ρ(C–H)_bipyam_, 773 (m). UV–vis: as nujol mull, λ (in nm): 602 (sh), 512, 420 (sh); in DMSO, λ (in nm) (ε, in M^−1^ cm^−1^): 615 (sh) (10), 515 (65), 430 (sh) (90), 382 (sh) (180), 323 (3400), 288 (7400). μ_eff_ at RT = 4.09 BM. The complex is soluble in MeOH, CH_3_CN, DMF and DMSO (Λ_M_ = 9 mho·cm^2^·mol^−1^, in 1 mM DMSO solution).

**[Co(flrx)_2_(phen)], 17:** For the synthesis of complex **17**, CoCl_2_·6H_2_O (59 mg, 0.25 mmol) was used as the MCl_2_ and phen (45 mg, 0.25 mmol) as the *N,N*′-donor. Orange product of **17** (125 mg, 50%) was collected after three months. *Anal*. calc. for [Co(flrx)_2_(phen)] (C_46_H_42_CoF_6_N_8_O_6_) (MW = 975.81). C: 56.62, H: 4.34, N: 11.48%; found: C: 56.40, H: 4.15, N: 11.29%. ESI–MS(+), *m*/*z*: found, 1045.93. IR (KBr disk), *ν*_max_ (cm^−1^): *ν*(C=O)_pyr_, 1623 (s); *ν*_asym_(COO), 1580 (s); *ν*_sym_(COO), 1371 (s); Δ*ν*(COO) = 209; ρ(C–H)_phen_, 731 (m). UV–vis: as nujol mull, λ (in nm): 600, 524, 423 (sh); in DMSO, λ (in nm) (ε, in M^–1^ cm^–1^): 610 (sh) (15), 532 (55), 430 (sh) (85), 382 (sh) (195), 332 (4100), 287 (9100). μ_eff_ at RT = 3.95 BM. The complex is soluble in MeOH, CH_3_CN, DMF and DMSO (Λ_M_ = 10 mho·cm^2^·mol^−1^, in 1 mM DMSO solution) and partially soluble in CH_2_Cl_2_.

**[Ni(flrx)_2_(bipyam)], 18:** For the synthesis of complex **18**, NiCl_2_·6H_2_O (59 mg, 0.25 mmol) was used as the MCl_2_ and bipyam (43 mg, 0.25 mmol) as the *N,N*′-donor. Green microcrystalline product of **18** (120 mg, 50%) was collected after two months. *Anal*. calc. for [Ni(flrx)_2_(bipyam)] (C_44_H_43_F_6_N_9_NiO_6_) (MW = 966.58). C: 58.68, H: 4.48, N: 13.04%; found: C: 58.45, H: 4.65, N: 12.79%. ESI–MS(–), *m*/*z*: found, 965.52; calc. for MW–1, 965.6.IR (KBr disk), *ν*_max_ (cm^–1^): *ν*(C=O)_pyr_, 1622 (s); *ν*_asym_(COO), 1579 (s); *ν*_sym_(COO), 1372 (s); Δ*ν*(COO) = 207; ρ(C–H)_bipyam_, 780 (m). UV–vis: as nujol mull, λ (in nm): 990, 620, 405 (sh); in DMSO, λ (in nm) (ε, in M^–1^ cm^–1^): 1000 (10), 607 (15), 415 (sh) (60), 324 (5600), 288 (8800); 10Dq = 10,000 cm^–1^, B = 705 cm^–1^, 10Dq/B = 14.2. μ_eff_ at RT = 3.05 BM. The complex is soluble in DMF and DMSO (Λ_M_ = 12 mho·cm^2^·mol^−1^, in 1 mM DMSO solution) and partially soluble in methanol.

**[Ni(flrx)_2_(phen)], 19:** For the synthesis of complex **19**, NiCl_2_·6H_2_O (59 mg, 0.25 mmol) was used as the MCl_2_ and phen (45mg, 0.25 mmol) as the *N,N*′-donor. Green microcrystalline product of **19** (125 mg, 50%) was collected after six weeks. *Anal*. calc. for [Ni(flrx)_2_(phen)] (C_46_H_42_F_6_N_8_NiO_6_) (MW = 975.59). C: 56.63, H: 4.34, N: 11.49%; found: C: 56.45, H: 4.11, N: 11.25%. ESI–MS(+), *m*/*z*: found, 996.28; calc. for MW+1 +H_2_O, 994.59. IR (KBr disk), *ν*_max_ (cm^−1^): *ν*(C=O)_pyr_, 1615 (s); *ν*_asym_(COO), 1578 (s); *ν*_sym_(COO), 1372 (s); Δ*ν*(COO) = 206; ρ(C–H)_phen_, 730 (m). UV–vis: as nujol mull, λ (in nm): 985, 610, 402 (sh); in DMSO, λ (in nm) (ε, in M^−1^ cm^−1^): 1000 (10), 605 (10), 410 (sh) (80), 333 (3800), 287 (8500); 10Dq = 10,000 cm^–1^, B = 728 cm^–1^, 10Dq/B = 13.7. μ_eff_ at RT = 2.85 BM. The complex is soluble in MeOH, CH_3_CN, DMF and DMSO (Λ_M_ = 8 mho·cm^2^·mol^−1^, in 1 mM DMSO solution) and partially soluble in CH_2_Cl_2_.

### 2.3. X-ray Crystal Structure Determination

An Agilent Technologies SuperNova Dual Diffractometer with Mo–Kα radiation (*λ* = 0.71073 Å) or Cu–Kα radiation (*λ* = 1.54184 Å) was used for single-crystal X-ray diffraction data collection at 150 K. CrysAlis Pro [37] was used for data processing. Structures were solved using direct methods with SHELXS or SHELXT program suites [38] and refined with SHELXL [39]. Anisotropic refinement was applied for all non-hydrogen atoms. Hydrogen atoms were readily located in difference Fourier maps and were subsequently treated as riding atoms in geometrically idealized positions unless otherwise noted. In the crystal structure of **6**·4H_2_O, water hydrate molecules O6–O8 were refined with fixed occupancy factors of 0.50, 0.40 and 0.60, respectively, and O9 was disordered over the inversion center with a 0.50:0.50 ratio. Hydrogen atoms on water molecules O4–O9 were not found in difference Fourier maps and were not included in the refinement. Atoms O8, O9, F2, C12 and C16 were refined with restrained *U*^ij^ components. In the crystal structure of **7**·2MeOH·4H_2_O, hydrogen atoms on water hydrate molecules O6–O8 were refined, restraining the bonding distances. In the crystal structure of **9**·9.5H_2_O, hydrogen atoms on water hydrate molecules O7, O9, O10 were refined, restraining the bonding distances. Hydrogen atoms on water molecules O8, O12–O19 were not found in difference Fourier maps and were not included in the refinement. Water hydrate molecules O13–O16 were refined with fixed occupancy factors of 0.50, and water hydrate molecules O17 and O19 with fixed occupancy factors of 0.30 and 0.20, respectively, and O18 was disordered over two positions with a refined occupancy ratio of 0.34:0.66. Atom O14 was refined with restrained *U*^ij^ components. Crystallographic data are listed in Table S1.

### 2.4. Study of the Biological Profiles of the Compounds

All the specific protocols and relevant equations involved in the in vitro study of the biological activity (antimicrobial activity, interaction with CT DNA and albumins) of the compounds are presented in the Supporting Information file (Section 1, Section 2 and Section 3).

## 3. Results

### 3.1. Synthesis and Characterization

**Scheme 1:**
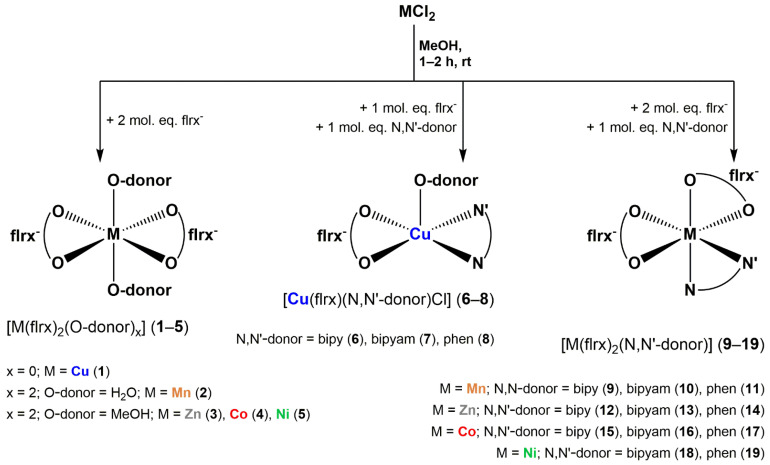
Synthetic route and numbering of complexes **1**–**19**.

To synthesize complexes **1**–**19** in good yield, appropriate metal(II) chlorides were reacted with deprotonated fleroxacin in methanol in the absence or presence of the chelating *N,N*′-donor ligands. Reactants in molar ratios MCl_2_:(flrx^–^) of 1:2 were used to form complexes **1**–**5**, CuCl_2_:(flrx^–^):(*N,N*′-donor) = 1:1:1 to form complexes **6**–**8** and MCl_2_:(flrx^–^):(*N,N*′-donor) = 1:2:1 to form complexes **9**–**19** (Scheme 1). The complexes were characterized by diverse physicochemical (elemental analysis, molar conductivity measurements) and spectroscopic (IR, UV–vis, ^1^H NMR, mass) techniques, RT magnetic measurements and single-crystal X-ray crystallography. Mass spectra (Figures S1–S7) in combination with the results of elemental analysis and X-ray diffraction data confirm the proposed structure of the synthesized complexes.

The values (Λ_M_) of the molar conductivity (1 mM DMSO solution) were found in the range 3–12 mho·cm^2^·mol^−1^ (for a 1:1 electrolyte, the Λ_M_ value should be ~70 mho·cm^2^·mol^−1^ [40]) and we may suggest that the obtained complexes are neutral and do not dissociate in DMSO solution.

Magnetic measurements of the complexes were carried out at room temperature. The derived values of μ_eff_ are in the range 1.78–1.86 BM for the copper(II) complexes, 5.90–6.05 BM for the manganese(II) complexes, 3.95–4.15 BM for the cobalt(II) complexes and 2.85–3.05 BM for the nickel(II) complexes. The μ_eff_ values are close to the spin-only values (=1.73 BM, 5.92 BM, 3.87 BM and 2.83 BM, respectively) at RT, further confirming the mononuclear structure of the complexes in solid state [41,42,43,44].

In the IR spectra of the complexes, the values of the Δ*ν*(COO) parameter (in the range 201–226 cm^−1^) are higher than the corresponding value in the potassium salt of fleroxacin (190 cm^−1^), supporting the monodentate binding mode of the carboxylato group of the fleroxacinato ligands [45,46], and subsequently leading to bidentate chelating binding through the pyridone oxygen and a carboxylato oxygen. Furthermore, the characteristic bands of the out-of-plane ρ(C–H)*_N,N_*_′-donor_ due to the presence of the corresponding α-diimine were also observed in complexes **6**–**19**, confirming the co-existence of the corresponding *N,N*′-donor co-ligands [45].

The stability of the complexes in solution was studied by UV–vis spectroscopy. The spectra of intact complexes were first recoded in solid state as nujol mull. Then, the complexes were dissolved in DMSO or buffer solutions, which were employed in biological experiments (150 mM NaCl and 15 mM trisodium citrate). The pH values of solutions were adjusted to 6–8 by dropwise addition of a HCl solution. The spectra of all complexes remained unchanged (no shift in λ_max_, or appearance of new bands), which confirms the integrity of the complexes in solution [26,27,28,29,30,31,32,33,34]. In particular, in the visible region of the spectra, the expected bands assigned to d–d transitions were observed for the copper(II) (one band at 645–675 nm), cobalt(II) (three bands at 610–630 nm, 515–540 nm and 430–435 nm) and Ni(II) (three bands at 995–1000 nm, 605–615 nm and 395–415 nm) complexes.

The ^1^H NMR spectra of the Zn(II)–flrx complexes in DMSO-*d*_6_ (Figures S8 and S9) are consistent with the obtained structures. All expected sets of signals related to the existence of the respective ligands in the corresponding compounds have been observed: seven signals for fleroxacin ligands and four for the *N,N*′-donor co-ligands. In the ^1^H NMR spectra of the Zn(II) complexes, the absence of a signal attributed to carboxylic hydrogen of free Hflrx proves its deprotonation upon binding to zinc(II) [26,47,48]. All signals were shifted slightly upon binding to zinc(II) ion. Any further signals due to dissociated ligands were not observed, proving the stability of the zinc complexes in DMSO solution [49,50].

### 3.2. Structure of the Complexes

Single crystals suitable for X-ray crystallography were obtained for four of the isolated complexes. In brief, the X-ray crystal structures of complexes **3**, **7** and **9** are new, while the structure of **6** is similar to the one reported by Xiao et al. in ref. [25].

#### 3.2.1. Description of the Crystal Structure of Complex [Zn(flrx)_2_(MeOH)_2_]·2MeOH (**3**·2MeOH)

**Figure 2:**
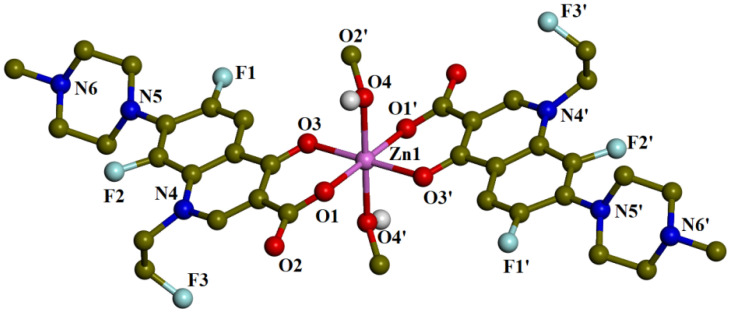
A drawing of the molecular structure of **3** with only the heteroatom labeling. Aromatic and methanol hydrogen atoms are omitted for clarity.

A drawing of the molecular structure of **3** is presented in Figure 2 and selected bond distances and angles are listed in Table 1.

Compound **3** is a centrosymmetric mononuclear complex, with the Zn(1) atom being on the inversion center. The fleroxacin ligands are deprotonated, being bidentately coordinated to the zinc ion via the pyridone oxygen O(3) and the carboxylate oxygen O(1), forming two six-membered chelate rings. The Zn atom has a slightly distorted octahedral geometry resulting from a ZnO_6_ coordination sphere formed by four oxygen atoms of fleroxacin ligands and two oxygen atoms of the methanol ligands. The Zn–O_carb_ (Zn(1)–O(1) = 2.0116(17) Å) are the shortest bond distances and the Zn–O_MeOH_ (Zn(1)–O(4) = 2.129(2) Å) are the longest bond distances in the coordination sphere.

#### 3.2.2. Description of the Crystal Structures of Complexes [Cu(flrx)(bipy)CI]·4H_2_O (**6**·4H_2_O) and [Cu(flrx)(bipyam)Cl]·2MeOH·4H_2_O (**7**·2MeOH·4H_2_O)

**Figure 3:**
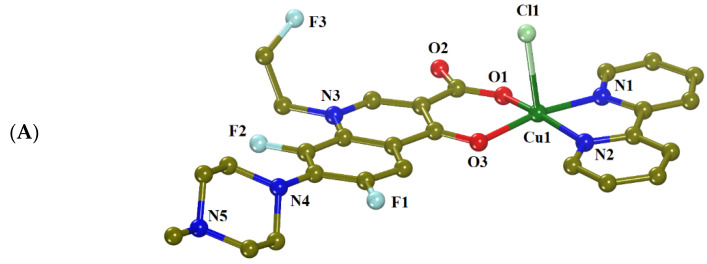

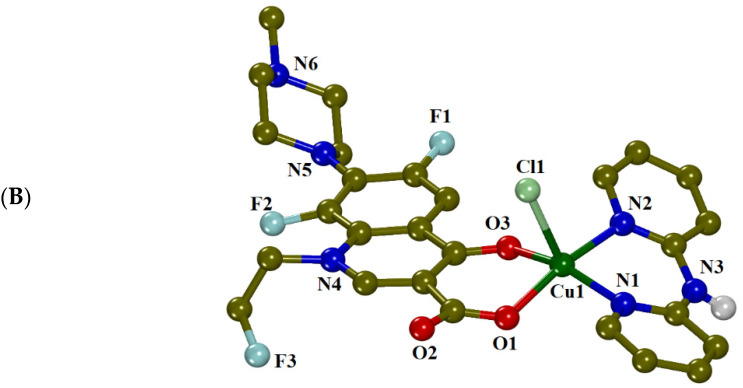
A drawing of the molecular structure of complex (**A**) **6** and (**B**) **7** with only the heteroatom labeling. Hydrogen atoms and solvate molecules are omitted for clarity.

The crystal structures of complexes **6** and **7** are presented in Figure 3 and selected bond distances and angles are summarized in Table 2. Since the structures present similarities, they are discussed together. There are four water solvate molecules in both structures and two additional methanol solvate molecules in complex **7**.

Both mononuclear complexes show a distorted square pyramidal geometry around the copper(II) ions, which are coordinated by the fleroxacinato and the α-diimine ligand in the equatorial plane. The coordination sphere is completed by a arboxyl ligand in the apical position to give neutral compounds. In both structures, the Cu(1)–O(1) are the shortest bond distances (in the range 1.904(3)–1.949(1) Å), the Cu(1)–Cl(1) bond distances (2.501(6)–2.528(2) Å) the longest ones and the Cu(1)–N bond distances (1.992(2)–2.004(6) Å) are slightly longer than the Cu(1)–O distances [1.904(3)–1.974(2) Å]. The distortion of the polyhedron is clearly visible and confirmed by the values of the trigonality and tetragonality indices τ [51] and T^5^ [41], respectively, (Table 2) and are the result of the non-planarity of the 2,2′-bipyridylamine ligand as well as the formation of the sterically more demanding six-membered ring upon coordination. Moreover, the distortion of the polyhedron is one of the highest observed in copper–quinolonato complexes with square–pyramidal geometry and a N_2_O_2_Cl coordination sphere reported thus far [12].

#### 3.2.3. Description of the Crystal Structure of [Mn(flrx)_2_(bipy)]·9.5H_2_O (**9**·9.5H_2_O)

**Figure 4:**
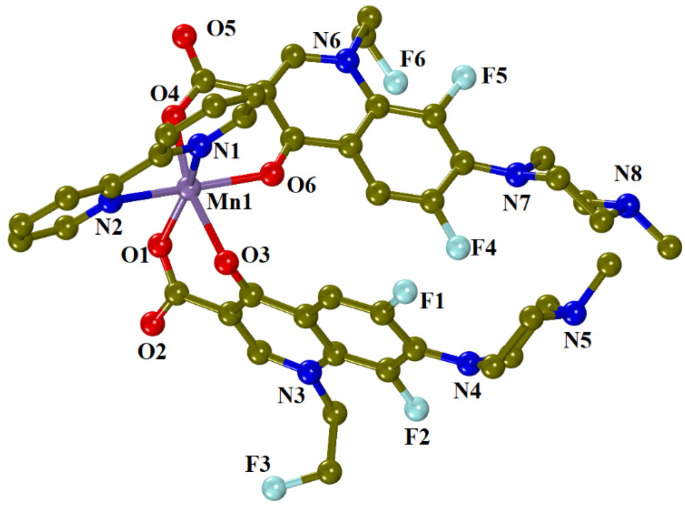
A drawing of the molecular structure of **9** with only the heteroatom labeling. Hydrogen atoms and solvate molecules are omitted for clarity.

The molecular structure of complex **9** is presented in Figure 4 and selected bond distances and angles are listed in Table 3.

Manganese complex **9** is mononuclear and the deprotonated fleroxacinato ligands are bound to the manganese(II) ion Mn1 in the usual bidentate chelating mode via the arboxyl oxygen and a arboxylate oxygen atoms. The octahedral environment of the manganese ion in which the pairs of chemically equivalent oxygen atoms of the quinolonato ligands are both in *cis* geometry is slightly distorted. This geometric isomerism is the rarest of the three possible geometries in metal-bis(quinolonato)-(α-diimine) systems and has been observed, to our knowledge, only in the nickel complex [Ni(flmq)_2_(bipy)_2_] (flmq = flumequinato) so far [52].

#### 3.2.4. Proposed Structures for the Remaining Complexes

By combining the results of spectroscopic and analytical experiments (IR, UV–vis, ^1^H NMR and mass spectroscopy, elemental analysis, molar conductivity and RT magnetic measurements) and literature data, we can propose, to a high degree of certainty, that all other complexes (**1**, **2**, **4**, **5**, **8** and **10**–**19**) are mononuclear and are neutral, resulting from the deprotonated fleroxacinato ligand(s), which are bound in a chelating mode to the metal ions in the absence or presence of the α-diimine ligands.

Complexes **2**, **4** and **5** have a similar structure to complex **3**, i.e., a six-coordinate central metal atom with a MO_6_ coordination sphere constituted of four coordinated fleroxacin oxygen atoms and two methanol oxygen atoms (most likely at *trans* positions), resulting in slightly distorted octahedral geometry around M. Complex **1** contains a four-coordinate Cu(II) ion with a CuO_4_ chromophore resulting from the four coordinated fleroxacin oxygen atoms. A similar structure was previously reported for complex [Cu(sf)_2_] (Hsf = the quinolone sparfloxacin) [53].

For complex **8**, the arrangement of the atoms (two fleroxacin oxygen atoms, two phen nitrogen atoms and the Cl atom) in a distorted square pyramidal environment around the five-coordinate copper is similar to that of complexes **6** and **7** and a series of previously reported [Cu(quinolone)(*N,N*′-donor)Cl] complexes [12,27,29,54,55].

For complexes **10**–**19,** we may propose analogous structures to complex **9**. Four of the six vertices of the octahedron around metal(II) are occupied by four oxygen atoms coming from the two fleroxacinato ligands and, in the remaining two positions, two nitrogen atoms of the respective *N,N*′-donor co-ligand form a six-membered chelate ring. Of course, the relevant arrangement of the arboxyl and the arboxylate oxygen atoms (*cis* or *trans*) around the central metal cannot be proposed since, in the literature, all possible arrangements have been reported [12,28,31,56,57].

### 3.3. Antimicrobial Activity

Three bacterial strains, one Gram(−) (*X. campestris*) and two Gram(+) (*S. aureus* and *B. subtilis*), were used in order to test the antimicrobial activity of the compounds. The MIC values of most compounds (Table 4) are low (4–32 μg/mL), indicating a potentiation of the antimicrobial activity in most cases, compared to the free quinolone, especially when the MIC values are compared in the molar scale. Most compounds appear to be more active against the Gram(+) bacterial strains (*S. aureus* and *B. subtilis*) than against the Gram(−) bacterial strain of *X. campestris*. In attempting to correlate the antimicrobial activity to structural elements, we propose that the chelating effect of the fleroxacinato ligands, the nature of the metal ions and the nature and the chelating effect of the *N,N*′-donor co-ligands [58,59] are the main factors resulting in such a high increase in potency; a discrete prevailing effect cannot be suggested, since almost all compounds, with some exceptions, have MIC values of the same order of magnitude.

### 3.4. Affinity of the Compounds for Albumins

**Figure 5:**
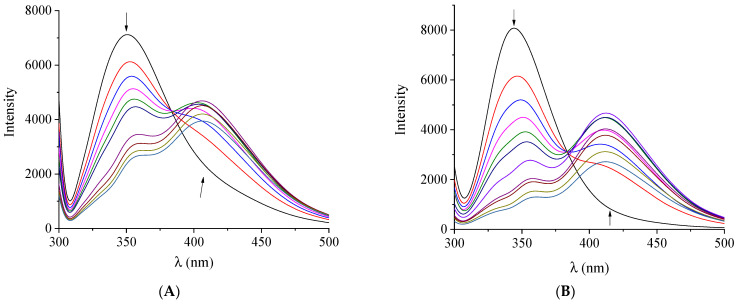
Fluorescence emission spectra (λ_excitation_ = 295 nm) of a buffer solution (150 mM NaCl and 15 mM trisodium citrate at pH 7.0) containing (**A**) HSA (3 μM) upon addition of increasing amounts of complex 1, and (**B**) BSA (3 μM) upon addition of increasing amounts of complex 2. The arrows show the changes in intensity upon increasing amounts of the complex.

Serum albumins (Sas) are the most abundant serum proteins and play a role in the circulatory system, primarily in the transport of drugs and other bioactive small molecules through the bloodstream [60]. Excitation of the solutions of BSA and HSA at 295 nm [61] leads to the appearance of an intense fluorescence emission band with λ_em,max_= 342 nm and 351 nm, respectively, which is attributed to tryptophan residues. An additional emission band in the range of 405–410 nm with the co-existence of an isoemissive point at 384 nm was also observed in the presence of the complexes; this emission band can be attributed to the presence of the complexes since it is also present in the fluorescence spectra of the free complexes upon excitation under the same experimental conditions. Therefore, the SA fluorescence emission spectra were corrected before further calculations (Figure 5). The inner-filter effect was also calculated with Equation (S1) [62] and it was found to affect the measurements only slightly. Addition of Hflrx and its complexes **1**–**19** into SA solutions (3 μM) resulted in a moderate quenching of the HSA fluorescence emission band at λ_em_ = 351 nm (Figure S10) and a more intense quenching of the BSA fluorescence emission band at λ_em_ = 342 nm (Figure S11). The observed quenching can be attributed to changes in the tryptophan environment of albumins, resulting from changes in their secondary structure, obviously due to the interaction of the compounds with SA [61].

The SA-quenching constants (k_q_) for the compounds (Table 5) were calculated with the Stern–Volmer quenching equation (Equations (S2) and (S3)). The k_q_ values are much higher than 10^10^ M^−1^s^−1^, indicating the existence of a static quenching mechanism [63] that proves the interaction of the compounds with the albumins. In all cases, the k_q_ constants of complexes **1**–**19** are much higher than that of free Hflrx, indicating that the formation of coordination compounds leads to increased affinity of the quinolones for the albumins. Of the compounds studied, complexes **3** and **15** have the highest k_q_ values for HSA and BSA, respectively (k_q(HSA),**3**_ = 2.26(±0.10) × 10^13^ M^−1^s^−1^ and k_q(BSA),**15**_ = 5.12(±0.17) × 10^13^ M^−1^s^−1^). The k_q_ constants of the complexes are within the range of previously reported values for metal(II)–quinolone complexes [26,27,28,29,30,31,32,33,34,52,56,57].

Albumin-binding constants (K) were calculated using the Scatchard equation (Equation (S4)). The K values for the complexes (Table 5) are relatively high (K_(HSA),**7**_ = 1.31(±0.05) × 10^5^ M^−1^ and K_(BSA),**15**_ = 2.84(±0.11) × 10^5^ M^−1^ are the highest values for each albumin) and are in the range of values reported for metal(II) complexes with quinolones as ligands [26,27,28,29,30,31,32,33,34,56,57]. The albumin-binding constants for the compounds are high enough to support albumin binding, leading to effective transport to their potential biological targets. On the other hand, they are significantly lower than the value of 10^15^ M^–1^ (this is the association constant of avidin with different compounds, which presents the strongest known noncovalent interactions), suggesting reversible binding potential for release upon reaching their biotargets [64].

### 3.5. Interaction with CT DNA

DNA is one of the most important molecules in all known organisms and many viruses [65]. It is the carrier of genetic information and is also a very suitable pharmacological target for drug development, as it can regulate functions such as transcription and regulation through specific protein interactions [66]. The best examples of approved drugs targeting DNA are used in cancer therapy, with the best known agent being cisplatin [67,68,69].

In general, metal complexes can interact with DNA through several types of interactions. Covalent interactions are the strongest and usually occur when the labile ligand(s) of the complex are replaced by a DNA-base nitrogen. A typical example is cisplatin binding to the N7 position of guanine bases [70]. Noncovalent binding can occur in the case of weaker interactions. Such interactions may be the consequence of various processes, e.g., π–π stacking between DNA base pairs leading to intercalation, Coulomb forces (electrostatic interactions), van der Waals forces, hydrogen bonding or hydrophobic interactions in groove binding [71]. As in our previous studies, we also sought to obtain more details about the interactions of isolated compounds with CT DNA. To this end, we have used UV–vis spectroscopy, viscosity measurements and cyclic voltammetry, as well as competitive binding studies with EB using fluorescence emission spectroscopy.

#### 3.5.1. DNA-Binding Studies by UV–vis Absorption Spectroscopy

The UV spectra of a DNA solution were recorded after successive additions of the compounds, and inversely the spectra of the complexes (5 × 10^−5^ – 10^−4^ M) in the presence of CT DNA in increasing amounts. The UV band of CT DNA with λ_max_ = 258–260 nm exhibited a slight hypochromism in the presence of the complexes (Figure S12), which was accompanied by a slight red shift, confirming the existence of the interaction.

**Figure 6:**
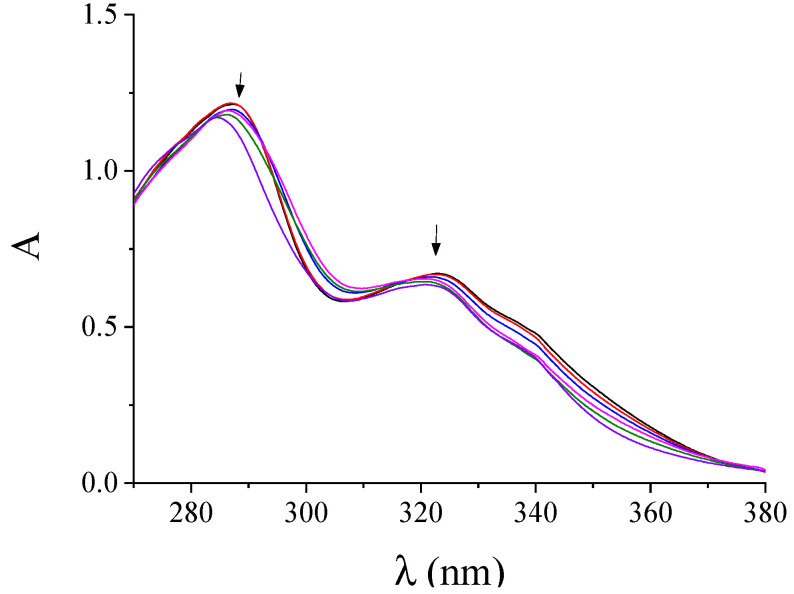
UV–vis spectra of DMSO solution of complex **16** (5 × 10^−5^ M) in the presence of increasing amounts of CT DNA. The arrows show the changes upon addition of increasing amounts of CT DNA.

In the UV–vis spectra of the complexes (shown for complex **16** in Figure 6), the bands attributed to the intra-ligand transitions showed a slight-to-moderate hypochromism in the presence of CT DNA (Table 6), further confirming the interaction. It should be noted that these spectroscopic features were not pronounced enough to suggest a certain mode of interaction between the complexes and CT DNA [71], and so further experiments (e.g., viscosity measurements, cyclic voltammetry) were performed.

The values of DNA-binding constant (K_b_) were calculated with the Wolfe–Shimer equation (Equation (S5)) and the [DNA]/(ε_A_ – ε_f_) versus [DNA] plots [72]. The K_b_ values of most complexes (Table 6) are significantly higher than that the K_b_ values of free fleroxacin and the classic intercalator EB (K_b(EB)_ = 1.23(±0.07) × 10^5^ M^−1^) [73]. Complex **6** exhibits the highest K_b_ constant (= 6.35(±0.02) × 10^7^ M^−1^) among the compounds studied and is among the highest K_b_ values reported for any metal(II)–quinolone complexes [26,27,28,29,30,31,32,33,34,47,48,52,56,57].

#### 3.5.2. Effect of the Complexes on the DNA-Viscosity

The changes in DNA-viscosity that occur upon the addition of a compound are usually indicative of the nature of the interaction between the compound and DNA, since relative DNA-viscosity is proportional to relative DNA-length [74]. Intercalation results in a longer distance between the base pairs, which leads to an increase in the relative DNA length, usually resulting in an increase in DNA-viscosity. Additionally, when the interaction occurs on the DNA surface (i.e., non-classical intercalation), a negligible decrease in the DNA-viscosity can be observed, since the relative DNA-length does not show a significant change.

**Figure 7:**
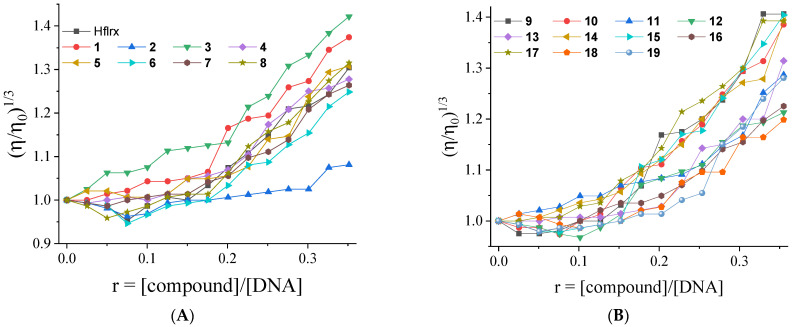
Relative viscosity (η/η_0_)^1/3^ of CT DNA (0.1 mM) in buffer solution (150 mM NaCl and 15 mM trisodium citrate at pH 7.0) in the presence of complexes (**A**) **1**–**8** and (**B**) **9**–**19** at increasing amounts (*r* = [compound]/[DNA] ratio= 0–0.35).

The viscosity of the CT DNA solution (0.1 mM) was measured after the addition of increasing amounts of complexes **1**–**19** (up to the value of *r* = 0.36) at room temperature (Figure 7). In almost all cases, a significant increase in DNA-viscosity was observed, which may indicate the existence of an intercalative interaction between the complexes and CT DNA [74].

#### 3.5.3. Study of the DNA-Interaction by Cyclic Voltammetry

Cyclic voltammetry was used to study the interaction of the complexes with CT DNA. An intercalative interaction will induce a positive shift for the electrochemical potential(s) of metal oxidation/reduction, whereas, in the case of electrostatic interaction, a negative shift in the potential(s) may occur [75]. The cyclic voltammograms of the complexes in a 1:2 DMSO:buffer solution (0.33 mM) were recorded in the absence and presence of the CT DNA solution (Figure S13). The cathodic (E_pc_) and anodic (E_pa_) potentials and corresponding shifts are summarized in Table 7. The predominant electrochemical feature for all complexes is a positive shift in the potential in the presence of CT DNA (ΔE_pc/a_ = (–30)–(+127) mV). Consequently, the intercalative nature of the interaction of the complexes with DNA can be inferred from the presented data.

In order to evaluate further the redox behavior of the complexes, the corresponding equilibrium constants were calculated by determining the ratio K_r_/K_ox_ in accordance to Equation (S6) [76], where K_ox_ and K_r_ are the DNA-binding constants for the oxidized and reduced form, respectively, of the metal ions. For all complexes, the K_r_/K_ox_ ratio is close to or above 1 (Table 7), showing the selective binding of DNA to the oxidized form of the complexes.

#### 3.5.4. Competitive Study with EB

Ethidium bromide is a typical marker of intercalation because it can intercalate between adjacent DNA bases with its planar phenanthridine ring. Such interaction with DNA leads to the formation of an EB–DNA adduct that exhibits an intense fluorescence emission band at 592 nm, when excited at 540 nm [61]. The intensity of this emission band in the presence of a compound that binds to DNA with comparable or greater potency than EB may be monitored to investigate the competition of the compound with EB for the DNA-intercalation site.

**Figure 8:**
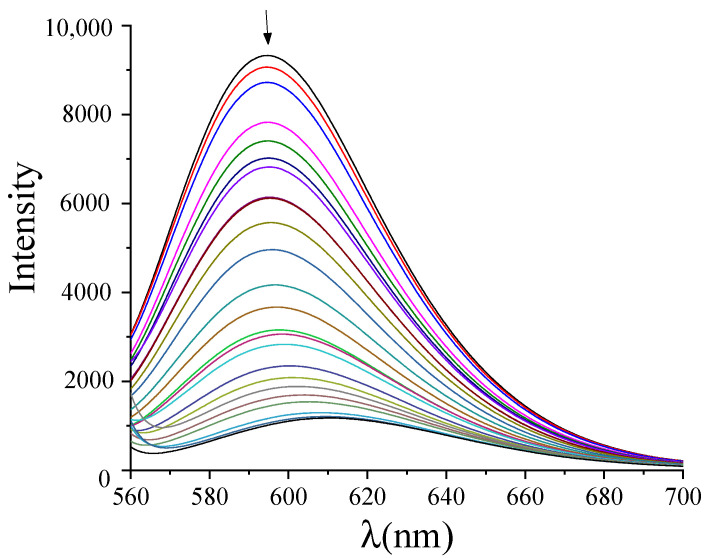
Fluorescence emission spectra (λ_exc_ = 540 nm) with a buffer solution (150 mM NaCl and 15 mM trisodium citrate at pH = 7.0) containing an EB–DNA adduct ([EB] = 20 μM, [DNA] = 26 μM) in the absence and in the presence of increasing amounts (up to *r* = 0.12) of [Zn(flrx)_2_(bipy)]. The arrow shows the changes in intensity upon increasing amounts of the complex.

The fluorescence emission spectra of the EB–DNA adduct ([EB] = 20 μM, [DNA] = 26 μM) in the absence and presence of the compounds were recorded at increasing amounts of the respective compounds. Addition of the complexes at different *r* values (shown representatively for complex **12** in Figure 8) resulted in a significant decrease in the intensity of the characteristic EB–DNA emission band at 592 nm. The observed attenuation of the fluorescence emission is up to 88.6% (Figure S14, Table 8) and may indicate the competition of the complexes with EB in binding to DNA. As a conclusion, an intercalative interaction of the complexes with CT DNA can be proposed [61].

The values of the Stern–Volmer constants (K_sv_) for the compounds were calculated using the linear Stern–Volmer equation (Equation (S2)) and were of the order of 10^5^ M^−1^ (Table 8), indicating tight binding of the complexes with DNA [61]. The K_sv_ values for the complexes are in the range found for a number of metal(II)–quinolone complexes, and complex **12** presents the highest K_sv_ value (K_sv_ = 7.25(±0.30) × 10^5^ M^−1^) among the compounds studied. In addition, the EB–DNA quenching constants (k_q_) for the compounds were calculated with Equation (S3), assuming τ_o_ = 23 ns as the fluorescence lifetime [77]. The values of k_q_ (Table 8) are much higher than the value of 10^10^ M^−1^s^−1^, indicating a static mechanism of EB–DNA fluorescence quenching induced by the compounds, which can result from the formation of a new adduct apparently consisting of each complex and DNA [61].

## 4. Conclusions

A series of some first-row transition metal(II) complexes with the quinolone fleroxacin have been synthesized in the absence or presence of *N,N*′-donors as co-ligands and characterized by various techniques including X-ray crystallography. The quinolone ligands in all complexes are bidentately coordinated to the metal(II) ion through the carboxylato and the pyridone oxygen atoms. The X-ray crystal structures of four of the nineteen complexes were characterized, representing all three types of the synthesized complexes.

The complexes are mainly soluble in DMSO and DMF and partially in solvents such as methanol, while they presented low aqueous solubility. In order to study the biological properties of the complexes, mixtures of DMSO with aqueous solutions of the biomacromolecules were used, where DMSO did not exceed 5% *v*/*v* in the final solution. In general, the use of DMSO is acceptable in such studies, although water is the most favorable solvent.

The antimicrobial activity of the compounds was evaluated against *X. campestris*, *S. aureus* and *B. subtilis* bacterial strains. In most cases, the complexes were more active than the free quinolone drug. Most complexes were effective against the Gram(+) bacteria *S. aureus* and *B. subtilis*, with the most active compounds showing MIC values lower than 5 μM.

The ability of the complexes to bind to bovine and human serum albumins was evaluated by fluorescence emission spectroscopy. The complexes bind tightly and reversibly to both albumins. Complexes [Cu(flrx)(bipyam)Cl], **7** and [Co(flrx)_2_(bipy)], **15** exhibited the highest affinity for HSA and BSA, respectively, as concluded after comparing the albumin-binding constants.

The interaction of the complexes with CT DNA probably occurs via intercalation, as indicated by the UV–vis titration studies, viscosity measurements and cyclic voltammetry experiments. On the basis of the DNA-binding constants, most of the reported complexes showed significantly high affinity for CT DNA, having K_b_ constants of the 10^6^−10^7^ M^−1^ magnitude, which are among the highest reported values for K_b_.

Overall, the metal(II)–fleroxacin complexes under study showed remarkable antimicrobial activity due to the binding of biologically relevant metal ions, which seems promising for further biological and pharmaceutical research studies.

## Figures and Tables

**Table 1:** Selected bond distances and angles for complex **3**.

Bond	Distance (Å)	Bond	Distance (Å)
Zn(1)–O(1)	2.012(2)	Zn(1)–O(3)	2.086(2)
Zn(1)–O(4)	2.129(2)		
**Bond Angle**	**(°)**	**Bond Angle**	**(°)**
O(1)–Zn(1)–O(3)	88.56(7)	O(1)–Zn(1)–O(3′)	91.44(7)
O(1)–Zn(1)–O(4′)	87.44(8)	O(3–Zn(1)–O(4)	92.95(8)
O(1)–Zn(1)–O(4)	92.56(8)	O(3–Zn(1)–O(4′)	87.05(8)

**Table 2:** Selected bond distances, bond angles and structural features for complexes **6** and **7**.

	[Cu(flrx)(bipy)Cl]	[Cu(flrx)(bipyam)Cl]
Bond	Distance (Å)	Distance (Å)
Cu(1)–O(1)	1.904(3)	1.949(1)
Cu(1)–O(3)	1.961(5)	1.974(2)
Cu(1)–N(1)	2.004(6)	1.992(2)
Cu(1)–N(2)	2.000(5)	1.998(2)
Cu(1)–Cl(1)	2.528(2)	2.501(6)
**Bond Angle**	**(°)**	**(°)**
Cl(1)–Cu(1)–N(1)	98.4(2)	105.07(5)
CI(1)–Cu(1)–N(2)	93.9(2)	100.54(5)
Cl(1)–Cu(1)–O(1)	99.7(1)	92.08(4)
Cl(1)–Cu(1)–O(3)	91.4(1)	96.37(4)
N(1)–Cu(1)–N(2)	81.1(2)	89.65(7)
N(1)–Cu(1)–O(1)	89.0(2)	88.58(6)
N(1)–Cu(1)–O(3)	169.4(2)	158.52(7)
N(2)–Cu(1)–O(1)	164.3(2)	167.28(6)
N(2)–Cu(1)–O(3)	94.2(2)	87.58(6)
O(1)–Cu(1)–O(3)	93.4(8)	89.46(6)
Trigonality index, τ ^a^	0.085	0.146
Tetragonality, T^5 b^	0.778	0.790

^a^ The trigonality index τ is determined based on bond angles in the coordination sphere. τ = (φ_1_ − φ_2_)/60°, where φ_1_ and φ_2_ are the largest angles in the coordination sphere, and its values may vary between 0 and 1 (τ = 0 corresponds to a perfect square pyramid and τ = 1 to a perfect trigonal bipyramid) [51]. ^b^ The tetragonality T^5^ is determined based on the M-ligand bond lengths. T^5^ = (mean in-plane distance M-L)/(mean out-of-plane distance M-L) [41].

**Table 3:** Selected bond distances and angles for complex **9**.

Bond	Distance (Å)	Bond	Distance (Å)
Mn(1)–O(1)	2.123(2)	Mn(1)–O(6)	2.147(2)
Mn(1)–O(3)	2.176(2)	Mn(1)–N(1)	2.263(2)
Mn(1)–O(4)	2.134(2)	Mn(1)–N(2)	2.254(2)
**Bond Angle**	**(°)**	**Bond Angle**	**(°)**
O(1)–Mn(1)–O(3)	82.58(6)	O(3)–Mn(1)–O(4)	161.81(7)
O(1)–Mn(1)–O(4)	93.68(7)	O(3)–Mn(1)–O(6)	81.78(6)
O(1)–Mn(1)–O(6)	101.33(7)	O(3)–Mn(1)–N(1)	88.80(7)
O(1)–Mn(1)–N(1)	158.87(7)	O(3)–Mn(1)–N(2)	91.90(7)
O(1)–Mn(1)–N(2)	90.15(7)	O(4)–Mn(1)–O(6)	81.52(7)
O(6)–Mn(1)–N(1)	95.33(7)	O(4)–Mn(1)–N(1)	99.89(7)
O(6)–Mn(1)–N(2)	165.98(7)	O(4)–Mn(1)–N(2)	105.95(7)
N(1)–Mn(1)–N(2)	71.91(7)		

**Table 4:** Antimicrobial activity of fleroxacin and its complexes **1**–**19** against *S. aureus, B. subtilis* and *X. campestris* expressed in MIC (in μg/mL and μM (values in parentheses)).

Compound	*S. aureus*	*B. subtilis*	*X. campestris*
Fleroxacin	4 (10.8)	4 (10.8)	8 (21.7)
[Cu(flrx)_2_], 1	8 (10.0)	8 (10.0)	8 (10.0)
[Cu(flrx)(bipy)Cl], 6	4 (6.4)	8 (12.8)	8 (12.8)
[Cu(flrx)(bipyam)Cl], 7	>16 (>25.0)	>16 (>25.0)	>32 (>50.0)
[Cu(flrx)(phen)Cl], 8	8 (12.3)	8 (12.3)	8 (12.3)
[Mn(flrx)_2_(MeOH)_2_], 2	4 (4.7)	4 (4.7)	16 (18.7)
[Mn(flrx)_2_(bipy)], 9	8 (8.4)	8(8.4)	16(16.9)
[Mn(flrx)_2_(bipyam)], 10	16 (16.6)	16 (16.6)	>32 (>33.2)
[Mn(flrx)_2_(phen)], 11	8 (8.2)	8 (8.2)	16 (16.4)
[Zn(flrx)_2_(MeOH)_2_], 3	8 (9.2)	8 (9.2)	32 (36.8)
[Zn(flrx)_2_(bipy)], 12	8 (8.3)	8 (8.3)	16 (16.7)
[Zn(flrx)_2_(bipyam)], 13	8 (8.2)	4 (4.1)	16 (16.4)
[Zn(flrx)_2_(phen)], 14	8 (8.1)	8 (8.1)	16 (16.3)
[Co(flrx)_2_(MeOH)_2_], 4	4 (4.6)	8 (9.3)	16 (18.6)
[Co(flrx)_2_(bipy)], 15	4 (4.2)	8 (8.4)	16 (16.8)
[Co(flrx)_2_(bipyam)], 16	8 (8.3)	8 (8.3)	16 (16.5)
[Co(flrx)_2_(phen)], 17	8 (8.0)	8 (8.0)	16 (16.0)
[Ni(flrx)_2_(MeOH)_2_], 5	4 (4.6)	4 (4.6)	16 (18.6)
[Ni(flrx)_2_(bipyam)], 18	8 (8.3)	8 (8.3)	32 (33.0)
[Ni(flrx)_2_(phen)], 19	8 (8.2)	8 (8.2)	16 (16.4)

**Table 5:** The (HSA-/BSA-) quenching (k_q_) and (HSA-/BSA-) binding constants (K) for fleroxacin and its complexes **1**–**19**.

Compound	k_q(HSA)_ (M^−1^s^−1^)	K_(HSA)_ (M^−1^)	k_q(BSA)_ (M^−1^s^−1^)	K_(BSA)_ (M^−1^)
Hflrx	2.69(±0.11) × 10^12^	6.59(±0.39) × 10^4^	6.19(±0.23) × 10^12^	3.28(±0.07) × 10^4^
[Cu(flrx)_2_], 1	1.09(±0.08) × 10^13^	8.39(±0.27) × 10^4^	1.87(±0.09) × 10^13^	7.19(±0.21) × 10^4^
[Cu(flrx)(bipy)Cl], 6	6.08(±0.06) × 10^12^	1.02(±0.03) × 10^5^	1.59(±0.07) × 10^13^	6.12(±0.33) × 10^4^
[Cu(flrx)(bipyam)Cl], 7	5.57(±0.18) × 10^12^	1.31(±0.05) × 10^5^	1.56(±0.04) × 10^13^	1.26(±0.05)×10^5^
[Cu(flrx)(phen)Cl], 8	4.08(±0.13) × 10^12^	3.45(±0.14) × 10^4^	1.04(±0.03) × 10^13^	5.03(±0.22) × 10^4^
[Mn(flrx)_2_(MeOH)_2_], 2	1.42(±0.04) × 10^13^	9.58(±0.39) × 10^4^	3.45(±0.16) × 10^13^	1.45(±0.04) × 10^5^
[Mn(flrx)_2_(bipy)], 9	8.09(±0.17) × 10^12^	6.36(±0.31) × 10^4^	2.45(±0.09) × 10^13^	1.39(±0.05) × 10^5^
[Mn(flrx)_2_(bipyam)], 10	8.01(±0.23) × 10^12^	8.40(±0.58) × 10^4^	1.20(±0.04) × 10^13^	6.46(±0.39) × 10^4^
[Mn(flrx)_2_(phen)], 11	1.03(±0.01) × 10^13^	1.01(±0.04) × 10^5^	1.65(±0.04) × 10^13^	1.09(±0.03) × 10^5^
[Zn(flrx)_2_(MeOH)_2_], 3	2.26(±0.10) × 10^13^	6.15(±0.25) × 10^4^	4.55(±0.21) × 10^13^	1.86(±0.08) × 10^5^
[Zn(flrx)_2_(bipy)], 12	1.24(±0.04) × 10^13^	1.04(±0.06) × 10^5^	1.74(±0.08) × 10^13^	7.89(±0.33) × 10^4^
[Zn(flrx)_2_(bipyam)], 13	1.23(±0.05) × 10^13^	3.69(±0.14) × 10^4^	3.37(±0.14) × 10^13^	3.60(±0.14) × 10^4^
[Zn(flrx)_2_(phen)], 14	1.10(±0.04) × 10^13^	6.16(±0.43) × 10^4^	1.61(±0.07) × 10^13^	5.56(±0.25) × 10^4^
[Co(flrx)_2_(MeOH)_2_], 4	6.05(±0.13) × 10^12^	7.68(±0.33) × 10^4^	1.34(±0.03) × 10^13^	1.02(±0.05) × 10^5^
[Co(flrx)_2_(bipy)], 15	1.56(±0.05) × 10^13^	8.13(±0.25)×10^4^	5.12(±0.17) × 10^13^	2.84(±0.11) × 10^5^
[Co(flrx)_2_(bipyam)], 16	9.35(±0.22) × 10^12^	9.61(±0.36) × 10^4^	1.98(±0.05) × 10^13^	1.44(±0.04) × 10^5^
[Co(flrx)_2_(phen)], 17	6.49(±0.21) × 10^12^	5.18(±0.32) × 10^4^	1.50(±0.03) × 10^13^	1.44(±0.03) × 10^5^
[Ni(flrx)_2_(MeOH)_2_], 5	1.09(±0.03) × 10^13^	5.76(±0.28) × 10^4^	2.37(±0.13) × 10^13^	1.75(±0.07) × 10^5^
[Ni(flrx)_2_(bipyam)], 18	6.94(±0.10) × 10^12^	7.13(±0.17) × 10^4^	1.56(±0.07) × 10^13^	4.77(±0.15) × 10^4^
[Ni(flrx)_2_(phen)], 19	6.67(±0.18) × 10^12^	6.65(±0.15) × 10^4^	1.39(±0.04) × 10^13^	8.01(±0.28) × 10^4^

**Table 6:** Spectral features of the UV–vis spectra of fleroxacin and its complexes **1**–**19** upon addition of DNA. UV–vis band (λ_max_, in nm) (percentage of hyper-/hypochromism (ΔA/A_0_, in %), blue/red shift of the λ_max_ (Δλ, in nm) and the corresponding DNA-binding constants (K_b_, in M^−1^).

Compound	Band (ΔA/A_0_^a^, Δλ ^b^)	K_b_ (M^–1^)
Hflrx	291 (−17, −6); 331 (+8, 0)	5.47(±0.38) × 10^4^
[Cu(flrx)_2_], 1	294 (−15, −7); 335 (−24, −10)	6.86(±0.16) × 10^6^
[Cu(flrx)(bipy)Cl], 6	294 (−21, −8); 314 (−38,−4); 332 (−11, −9)	6.35(±0.02) × 10^7^
[Cu(flrx)(bipyam)Cl], 7	294 (−18, −12); 316 (−8, 0)	4.07(±0.28) × 10^6^
[Cu(flrx)(phen)Cl], 8	293 (−31, −20); 326 (+1, −2)	9.64(±0.31) × 10^5^
[Mn(flrx)_2_(MeOH)_2_], 2	291 (−22, −6); 322 (−2, +4)	1.54(±0.23) × 10^6^
[Mn(flrx)_2_(bipy)], 9	286 (+4, −1); 334 (−11, −10)	1.08(±0.06) × 10^6^
[Mn(flrx)_2_(bipyam)], 10	271 (+3, +1); 317 (−5, −1)	2.34(±0.12) × 10^4^
[Mn(flrx)_2_(phen)], 11	288 (−7, −4); 331 (−8, −6)	9.69(±0.12) × 10^5^
[Zn(flrx)_2_(MeOH)_2_], 3	334 (−17, −1)	9.15(±0.30) × 10^5^
[Zn(flrx)_2_(bipy)], 12	290 (−15, −4); 332 (−7, −6)	1.02(±0.01) × 10^7^
[Zn(flrx)_2_(bipyam)], 13	291.2 (−17, −7); 322 (−5, −1)	1.11(±0.10) × 10^6^
[Zn(flrx)_2_(phen)], 14	288 (−1, −1); 332 (−10, −7)	1.03(±0.14) × 10^6^
[Co(flrx)_2_(MeOH)_2_], 4	289 (−8, −4); 330 (−7, −6)	9.74(±0.11) × 10^5^
[Co(flrx)_2_(bipy)], 15	288 (−3, −3); 331 (−8, −7)	1.35(±0.11) × 10^6^
[Co(flrx)_2_(bipyam)], 16	287 (−5, −3); 323 (−6, −2)	4.75(±0.24) × 10^5^
[Co(flrx)_2_(phen)], 17	286.6 (−2,− 3); 333 (0, −9)	1.73(±0.18) × 10^6^
[Ni(flrx)_2_(MeOH)_2_], 5	289 (−8, −3); 336 (−21, −10)	1.43 (±0.19) × 10^6^
[Ni(flrx)_2_(bipyam)], 18	290 (−10, −5); 323 (−6, −3)	1.65 (±0.11) × 10^6^
[Ni(flrx)_2_(phen)], 19	288.4 (−7, −4); 333 (−15, −8)	1.89 (±0.11) × 10^6^

^a^ “+” denotes hyperchromism and “−” denotes hypochromism. ^b^ “+” denotes red shift and “−” denotes blue shift.

**Table 7:** Cathodic and anodic potentials (in mV) for the redox couple M(II)/M(I) of the fleroxacin complexes in a 1:2 DMSO/buffer solution in the absence or presence of CT DNA. Ratio of equilibrium binding constants, K_r_/K_ox_.

Complex	E_pc(f)_^a^	E_pc(b)_^b^	ΔE_pc_^c^	E_pa(f)_^a^	E_pa(b)_^b^	ΔE_pa_^c^	K_r_/K_ox_
[Cu(flrx)_2_], **1**	−725	−735	−10	−504	−462	+42	1.31
[Cu(flrx)(bipy)Cl], **6**	−710	−740	−30	−450	−323	+127	2.28
[Cu(flrx)(bipyam)Cl], **7**	−729	−710	+19	−426	−300	+126	3.41
[Cu(flrx)(phen)Cl], **8**	−725	−720	+5	−483	−406	+77	2.00
[Mn(flrx)_2_(MeOH)_2_], **2**	−684	−678	+6	−455	−466	−11	0.96
[Mn(flrx)_2_(bipy)], **9**	−701	−684	+17	−475	−495	−20	0.97
[Mn(flrx)_2_(bipyam)], **10**	−706	−695	+11	−495	−508	−13	0.98
[Mn(flrx)_2_(phen)], **11**	−706	−700	+6	−501	−499	+2	1.07
[Co(flrx)_2_(MeOH)_2_], **4**	−724	−735	−11	−483	−455	+28	1.15
[Co(flrx)_2_(bipy)], **15**	−701	−695	+6	−533	−541	−8	0.98
[Co(flrx)_2_(bipyam)], **16**	−729	−741	−12	−466	−445	+21	1.08
[Co(flrx)_2_(phen)], **17**	−695	−689	+6	−523	−518	+5	1.10
[Ni(flrx)_2_(MeOH)_2_], **5**	−706	−701	+5	−495	−501	−6	0.99
[Ni(flrx)_2_(bipyam)], **18**	−695	−689	+6	−523	−512	+11	1.15
[Ni(flrx)_2_(phen)], **19**	−706	−718	−12	−489	−460	+29	1.15

^a^ Ep_c/a_ in DMSO/buffer in the absence of CT DNA (Ep_c/a(f)_). ^b^ Ep_c/a_ in DMSO/buffer in the presence of CT DNA (Ep_c/a(b)_). ^c^ ΔEp_c/a_ = Ep_c/a(b)_ − Ep_c/a(f)_.

**Table 8:** Percentage of EB–DNA fluorescence quenching (ΔI/Io, in %), Stern–Volmer constant (K_SV_, in M^−1^) and the quenching constant (k_q_, in M^−1^s^−1^) for fleroxacin and its complexes **1**–**19**.

Compound	ΔI/I_O_ (%)	K_sv_ (M^–1^)	k_q_ (M^−1^s^−1^)
Hflrx	65.8	4.64(±0.14) × 10^5^	2.02(±0.06) × 10^13^
[Cu(flrx)_2_], **1**	62.2	2.18(±0.06) × 10^5^	9.47(±0.03) × 10^12^
[Cu(flrx)(bipy)Cl], **6**	61.9	2.03(±0.05) × 10^5^	8.84(±0.21) × 10^12^
[Cu(flrx)(bipyam)Cl], **7**	62.7	3.44(±0.06) × 10^5^	1.50(±0.03) × 10^13^
[Cu(flrx)(phen)Cl], **8**	66.4	3.25(±0.08) × 10^5^	1.41(±0.03) × 10^13^
[Mn(flrx)_2_(MeOH)_2_], **2**	74.4	6.07(±0.23) × 10^5^	2.64(±0.10) × 10^13^
[Mn(flrx)_2_(bipy)], **9**	73.8	3.31(±0.07) × 10^5^	1.44(±0.03) × 10^13^
[Mn(flrx)_2_(bipyam)], **10**	81.1	5.92(±0.14) × 10^5^	2.57(±0.06) × 10^13^
[Mn(flrx)_2_(phen)], **11**	76.7	1.83(±0.03) × 10^5^	7.94(±0.14) × 10^12^
[Zn(flrx)_2_(MeOH)_2_], **3**	85.9	6.07(±0.17) × 10^5^	2.64(±0.07) × 10^13^
[Zn(flrx)_2_(bipy)], **12**	88.6	7.25(±0.30) × 10^5^	3.15(±0.13) × 10^13^
[Zn(flrx)_2_(bipyam)], **13**	81.8	6.51(±0.19) × 10^5^	2.84(±0.08) × 10^13^
[Zn(flrx)_2_(phen)], **14**	77.9	3.03(±0.05) × 10^5^	1.32(±0.02) × 10^13^
[Co(flrx)_2_(MeOH)_2_], **4**	64.1	1.36(±0.03) × 10^5^	5.93(±0.14) × 10^12^
[Co(flrx)_2_(bipy)], **15**	80.4	4.69(±0.09) × 10^5^	2.04(±0.04) × 10^13^
[Co(flrx)_2_(bipyam)], **16**	70.7	3.77(±0.12) × 10^5^	1.64(±0.05) × 10^13^
[Co(flrx)_2_(phen)], **17**	69.8	3.56(±0.10) × 10^5^	1.55(±0.05) × 10^13^
[Ni(flrx)_2_(MeOH)_2_], **5**	56.9	2.33(±0.08) × 10^5^	1.01(±0.03) × 10^13^
[Ni(flrx)_2_(bipyam)], **18**	59.3	3.02(±0.13) × 10^5^	1.31(±0.05) × 10^13^
[Ni(flrx)_2_(phen)], **19**	68.1	3.18(±0.14) × 10^5^	1.38(±0.05) × 10^13^

## Data Availability

Supplementary data associated with this article can be found in the online version.

## Supplementary Materials

CCDC 2143072–2143075 contain the supplementary crystallographic data for compounds **3**, **6**, **7** and **9**, respectively. These data can be obtained free of charge via www.ccdc.cam.ac.uk/conts/retrieving.html (accessed on 12 April 2022) (or from the Cambridge Crystallographic Data Centre, 12 Union Road, Cambridge CB21EZ, UK; Fax: (+44)-1223-336-033; or deposit@ccde.cam.ac.uk). Supplementary data associated with this article can be found in the online version, at https://www.mdpi.com/article/10.3390/pharmaceutics14050898/s1. Cif files for compounds **3**, **6**, **7** and **9**. Experimental protocols: S1 Antimicrobial activity; S2 Interaction with serum albumins; S3 Interaction with CT DNA. Table S1: Crystal determination data for complexes. Figures S1–S7: Mass spectra of the complexes. Figures S8 and S9: ^1^H NMR spectra of Hflrx and its Zn(II) complexes. Figure S10: Plot of % relative intensity has fluorescence emission band at λ_em,max_ = 351 nm (I/I_o_, %) versus *r* (*r* = [complex]/[HSA]) in the presence of the compounds. Figure S11: Plot of % relative intensity BSA fluorescence emission band at λ_em,max_ = 342 nm (I/I_o_, %) versus *r* (*r* = [complex]/[BSA]) in the presence of the compounds. Figure S12. UV–vis spectra of a buffer solution of CT DNA upon addition of increasing amounts of the compounds. Figure S13. Cyclic voltammograms of the complexes in the absence or presence of CT DNA. Figure S14. Plots of EB–DNA relative fluorescence emission intensity at λ_emission_ = 592 nm (%) versus *r* (*r* = [complex]/[DNA]) in the presence of the compounds. References [78,79] are cited in the supplementary materials.

## Abbreviations

*B. subtilis* = *Bacillus subtilis* ATCC 6633; bipy = 2,2′-bipyridine; bipyam = 2,2′-bipyridylamine; br = broad; BSA = bovine serum albumin; CT = calf-thymus; EB = 3,8-diamino–5-ethyl-6-phenyl-phenanthridinium bromide, ethidium bromide; E_pa_ = anodic potential; E_pc_ = cathodic potential; flrx^–^ = anion of fleroxacin; Hflrx = fleroxacin, 6,8-difluoro-1-(2-fluoroethyl)-7-(4-methylpiperazin-1-yl)-4-oxoquinoline-3-carboxylic acid; HSA = human serum albumin; K = SA-binding constant; K_b_ = DNA-binding constant; K_ox_ = DNA-binding constant for the oxidized form; k_q_ = quenching constant; K_red_ = DNA-binding constant for the reduced form; K_SV_ = Stern–Volmer constant; m = medium; MIC = minimum inhibitory concentration; phen = 1,10-phenanthroline; r = [compound]/[DNA] or [compound]/[SA] ratio; RT = room temperature; *S. aureus = Staphylococcus aureus* ATCC 6538; s = strong; SA = serum albumin; sh = shoulder; *X. campestris = Xanthomonas campestris* ATCC 1395.
