# Supplementary material for: Metal(II) Complexes of the Fluoroquinolone Fleroxacin: Synthesis, Characterization and Biological Profile
Source: Pharmaceutics. 2022 Apr 20;14(5):898. doi: 10.3390/pharmaceutics14050898 (PMC9144902; doi:10.3390/pharmaceutics14050898)
Supplement: Supplementary file 1 [file pharmaceutics-14-00898-s001.zip › pharmaceutics-1608171-supplementary.pdf]

Metal(II) Complexes of the Fluoroquinolone Fleroxacin: Synthesis,
Characterization and Biological Profile

Alexandra Kostelidou,[a] Franc Perdih,[b] Jakob Kljun,[b] Foteini Dimou,[c] Stavros Kalogiannis,[c] Iztok Turel,[b],* George Psomas,[a],*

[a] *Department of General and Inorganic Chemistry, Faculty of Chemistry, Aristotle University of Thessaloniki, GR–54124 Thessaloniki, GREECE.*

[b] *Faculty of Chemistry and Chemical Technology, University of Ljubljana, Vecna pot 113, 1000 Ljubljana, SLOVENIA.*

[c]*Department of Nutritional Sciences and Dietetics, International Hellenic University, Sindos, Thessaloniki, GREECE.*

Supplementary material

To evaluate the *in vitro* biological activity (antimicrobial activity, interaction with CT DNA and BSA and HSA) of the compounds, they were dissolved in DMSO (1 mM), due to their low aqueous solubility. The studies were conducted in the presence of aqueous buffer solutions of the biomacromolecules, where mixing of each solution never exceeded 5% DMSO (v/v) in the final solution. The effect of DMSO on the data was assessed *via* control experiments. Minimal or no changes were observed in the spectra of the albumins or CT DNA and appropriate corrections were performed, where necessary.

In brief, the potential antimicrobial activity of the compounds was evaluated by determining the MIC values against three bacterial species, one Gram–(–) (*X. campestris*)and two Gram–(+)(*S. aureus* and *B. subtilis*) microorganisms. The interaction of the compounds with CT DNA was thoroughly examined by UV–vis spectroscopy, viscosity measurements and cyclic voltammetry, as well as *via* competitive studies with EB by fluorescence emission spectroscopy. The BSA– and HSA–binding was studied through tryptophan fluorescence quenching experiments.

S1. Antimicrobial activity

* Corresponding authors' e–mails:

Iztok Turel: Iztok.Turel@fkkt.uni-lj.si ; George Psomas: gepsomas@chem.auth.gr

The antimicrobial activity of the compounds was evaluated by determining their respective MIC values towards one Gram–(–) (*Xanthomonas campestris* ATCC 1395 (*X. campestris*)) and two Gram–(+) (*Staphylococcus aureus* ATCC 6538 (*S. aureus*) and *Bacillus subtilis* ATCC 6633 (*B. subtilis*)) bacterial species. Cultures of these microbial strains were grown on a rich selective agar medium and stored at 4°C. The selective media used were Nutrient Agar or Broth for *B. subtilis* and *S. aureus*, and Yeast Mold Agar or Broth for *X. campestris.* Cells picked from the surface of the stored cultures were used to initiate liquid pre–cultures of the same selective medium at an initial turbidity of roughly 1 McFarland unit. Pre–cultures were incubated for 24 h in a rotary shaking incubator and subsequently they were used to inoculate the test cultures used for the determination of MIC at an initial turbidity of 0.5 McFarland units. The test cultures consisted of Mueller–Hinton broth (Deben Diagnostics Ltd) containing different concentrations of the compounds. Different concentrations were achieved as follows: the compounds were freshly dissolved in DMSO to a concentration of 1 mg/mL and they were diluted with DMSO, using the method of progressive double dilution. Therefore, working solutions with decreasing concentrations of the compounds under investigation were achieved. The working solutions were subsequently diluted to the final desired concentration by addition to the growth medium at a proportion of 2:98. MIC values were determined as the lowest concentrations of the tested compounds that inhibited visible growth of each respective organism after a 24–h incubation [S1]. Bacterial growth was determined by measuring the turbidity of appropriately diluted cultures at 600 nm with reference to equally diluted sterile growth medium and the inhibition achieved was calculated by comparing the turbidity of each culture to the average of the turbidity of three non–inhibited cultures. All test cultures were grown in triplicates and for the determination of MIC, growth had to be inhibited in at least two cultures of the triplicate. Incubation temperature at all stages was 37°C except for *X. campestris* that was cultivated at 28°C [S2].

S2. Interaction with serum albumins

The albumin–binding study for the compounds was carried out by fluorescence emission quenching experiments using BSA (3 μM) or HSA (3 μM) in buffer solution (15 mM trisodium citrate and 150 mM NaCl at pH 7.0). The tested compounds were used as quenchers with gradually increasing concentrations to monitor the quenching of the emission intensity of tryptophan residues of BSA at 345 nm or HSA at 340 nm [S3]. The fluorescence emission spectra were recorded between 300–500 nm with excitation wavelength of 295 nm. All the experiments were conducted at room temperature. The fluorescence spectra of the compounds were recorded under the same experimental conditions and presented a low–intensity emission band in the region 405–410 nm.

Consequently, the SA fluorescence emission spectra were modified properly, by subtracting the spectra of the compounds, and quantitative studies followed.

The extent of the inner–filter effect can be roughly estimated with the following formula:

$$I_{corr} = I_{meas} \times 10^{\frac{\varepsilon(\lambda_{exc})cd}{2}} \times 10^{\frac{\varepsilon(\lambda_{em})cd}{2}} \qquad \text{(eq. S1)}$$

where I_{corr}= corrected intensity, I_{meas}= the measured intensity, c = the concentration of the quencher, d = the cuvette length (1 cm), $\varepsilon(\lambda_{exc})$ and $\varepsilon(\lambda_{em})$ = the ε of the quencher at the excitation and the emission wavelength, respectively, as calculated from the UV–vis spectra of the complexes [S4].

The interaction of the quencher (i.e. compounds) with serum albumins was studied through the Stern–Volmer and Scatchard equations [S3] and corresponding graphs. The values of the respective Stern–Volmer constant (K_{SV}), the quenching constant (k_q), the SA–binding constant (K) and the number of binding sites per albumin (n) were calculated.

According to Stern–Volmer quenching equation [S3] (eq. S2),

$$\frac{Io}{I} = 1 + k_q \tau_0 [Q] = 1 + K_{SV}[Q] \qquad \text{(eq. S2)}$$

where I_0 = the initial tryptophan fluorescence intensity of SA, I = the tryptophan fluorescence intensity of SA after the addition of the quencher, k_q = the quenching rate constants of SA, K_{SV} = the dynamic quenching constant, τ_0 = the average lifetime of SA without the quencher, [Q] = the concentration of the quencher), the Stern–Volmer constant (K_{SV}) can be obtained by the slope of the diagram I_0/I *versus* [Q]. Taking $\tau_0 = 10^{-8}$ s as fluorescence lifetime of tryptophan in SA [S5], the quenching constant (k_q) is calculated from equation S3.

$$K_{SV} = k_q \tau_o \qquad \text{(eq. S3)}$$

From the Scatchard equation (eq. S4) [S3]:

$$\frac{\Delta I / I_0}{[Q]} = nK - K\frac{\Delta I}{I_0} \qquad \text{(eq. S4)}$$

where n = the number of binding sites per albumin and K = the SA–binding constant. The value of K is calculated from the slope in plots ($\Delta I/I_0$)/[Q] *versus* ($\Delta I/I_0$) and n is given by the ratio of y intercept to the slope [S6].

S3. Interaction with CT DNA

The interaction of fleroxacin and its complexes **1–19** with CT–DNA was investigated by UV–vis spectroscopy, viscosity measurements, cyclic voltammetry and fluorescence emission spectroscopy studies.

S3.1 Binding study with CT DNA by UV–vis spectroscopy

UV–vis spectroscopy was used for the evaluation of the interaction of the compounds with CT DNA, and specifically the possible binding mode of the compounds to CT DNA. Control experiments with DMSO were performed and no changes in the spectra of CT DNA were observed.

In order to determine the binding mode, the UV–vis spectra of the compounds were recorded for a constant concentration (5×10^{-5}–10^{-4} M) at the corresponding λ_{max} with increasing concentrations of CT DNA for diverse *r* (*r* = [complex]/[DNA]) values. Effective use of the changes in the absorbance of the UV–vis spectra was made and the DNA–binding constants (K_b, M^{-1}) of the compounds were calculated by the Wolfe–Shimer equation (eq. S5) [S7] and the plots [DNA]/(ε_A–ε_f) *versus* [DNA]:

$$\frac{[\mathrm{DNA}]}{(\varepsilon_A - \varepsilon_f)} = \frac{[\mathrm{DNA}]}{(\varepsilon_b - \varepsilon_f)} + \frac{1}{K_b(\varepsilon_b - \varepsilon_f)} \qquad \text{(eq. S5)}$$

where [DNA] = the concentration of DNA in base pairs, ε_f = the extinction coefficient for the free compound at the corresponding λ_{max}, $\varepsilon_A = A_{obsd}$/[compound], and ε_b = the extinction coefficient for the compound in the fully bound form. K_b is given by the ratio of slope to the y intercept in plots [DNA]/(ε_A–ε_f) *versus* [DNA].

S3.2 CT DNA–binding studies by viscosity measurements

The viscosity of DNA (0.1 mM) in buffer solution was measured in the absence and presence of increasing amounts of the compounds. The experiments were executed at room temperature and the measurements are devised in a plot $(\eta/\eta_0)^{1/3}$ *versus* r, where η = the viscosity of DNA in the presence of the compound, and η_0 = the viscosity of DNA in buffer solution.

S3.3 Study of the DNA–interaction by cyclic voltammetry

Cyclic voltammetry can be also used in order to calculate the corresponding equilibrium constant for the redox process. The ratio of the DNA–binding constants for the reduced (K_r) and oxidized forms (K_{ox}) of the complexes (K_r/K_{ox}) was calculated according to eq. S6:

$$\Delta E^o = E^o_{(b)} - E^o_{(f)} = 0.059 \times \log \frac{K_r}{K_{ox}} \qquad \text{(eq. S6)}$$

where $E^o_{(b)}$ and $E^o_{(f)}$ are the formal potentials of M(II)/M(I) redox couple in the fully bound and free complexes, respectively. K_{ox} and K_r are the binding constants for the binding of the oxidized and reduced species to DNA, respectively [S8].

S3.4 EB–displacement studies

In order to determine and confirm the DNA–binding mode of the compounds, a competitive study with EB as an intercalating marker is performed by fluorescence emission spectroscopy. Therefore, the EB–displacing ability of the compounds from its EB–DNA conjugate was examined.

The DNA–EB adduct was prepared by addition of 20 μM EB and 26 μM CT DNA in buffer solution (150 mM NaCl and 15 mM trisodium citrate at pH 7.0). The potential intercalation of the compounds between the DNA–bases was studied by the addition of a certain amount of the compound solution into the EB–DNA adduct solution. The influence of the compounds on the EB–DNA solution was monitored through the changes of the fluorescence emission spectra at excitation wavelength (λ_{ex}) at 540 nm [S3]. The tested compounds do not show any significant fluorescence at room temperature in solution or in the presence of DNA, under the same experimental conditions (λ_{ex} = 540 nm). Bearing that in mind, the observed quenching of the EB–DNA solution is evidently associated to the displacement of EB from its EB–DNA adduct.

The quenching efficiency (K_{SV}) for each compound was assessed according to the Stern–Volmer equation: $\frac{Io}{I} = 1 + k_q \tau_0 [Q] = 1 + K_{SV}[Q]$, (eq. S2) [S3] where I_0 and I = the fluorescence emission intensities of EB–DNA in the absence and presence of the quencher, respectively, [Q] = the concentration of the quencher (i.e. compounds). K_{SV} is obtained from the Stern–Volmer plots by the slope of the diagram I_0/I *versus* [Q]. Taking τ_0 = 23 ns as the fluorescence lifetime of the EB–DNA system [S5], the EB–DNA quenching constants (k_q) of the compounds can be determined according to eq. S3: $K_{SV} = k_q \tau_o$.

References

[S1] Andrews, J.M. Determination of minimum inhibitory concentrations. *J. Antimicr. Chemother.* **2001**, *48*(S1), 5–16.

[S2] Irgi, E.P.; Geromichalos, G.D.; Balala, S.; Kljun, J.; Kalogiannis, S.; Papadopoulos, A.; Turel, I.; Psomas, G. Cobalt(II) complexes with quinolone antimicrobial drug oxolinic acid: Structure and biological perspectives. *RSC Adv.* **2015**, *5,* 36353–36367.

[S3] J.R. Lakowicz, *Principles of Fluorescence Spectroscopy*, Plenum Press, New York, third ed., **2006**.

[S4] Stella, L.; Capodilupo, A.L.; Bietti, M. A reassessment of the association between azulene and [60]fullerene. Possible pitfalls in the determination of binding constants through fluorescence spectroscopy. *Chem. Commun.* **2008**. 4744–4746.

[S5] Heller, D.P.; Greenstock, C.L. Fluorescence lifetime analysis of DNA intercalated ethidium bromide and quenching by free dye. *Biophys. Chem.* **1994**, *50*, 305–312.

[S6] Wang, Y.; Zhang, H.; Zhang, G.; Tao, W.; Tang, S. Interaction of the flavonoid hesperidin with bovine serum albumin: A fluorescence quenching study. J. Lumin. 126 (2007) 211–218.

[S7] Wolfe, A.; Shimer, G.; Meehan, T. Polycyclic Aromatic Hydrocarbons Physically Intercalate into Duplex Regions of Denatured DNA. *Biochemistry* **1987**, *26*, 6392–6396.

[S8] Carter, M.T.; Rodriguez, M.; Bard, A.J. Voltammetric studies of the interaction of metal chelates with DNA. 2. Tris-chelated complexes of cobalt(III) and iron(II) with 1,10-phenanthroline and 2,2'-bipyridine. *J. Am. Chem. Soc*. **1989**, *111*, 8901–8911.

Tables

Table S1. Crystal determination data for complexes $[Zn(flrx)_2(MeOH)_2]\cdot 2MeOH$ (**3**·2MeOH), $[Cu(flrx)(bipy)Cl]\cdot 4H_2O$ (**6**·$4H_2O$), $[Cu(flrx)(bipyam)Cl]\cdot 2MeOH\cdot 4H_2O$ (**7**·$2MeOH\cdot 4H_2O$) and $[Mn(flrx)_2(bipy)]\cdot 9.5H_2O$ (**9**·$9.5H_2O$).

	**3**·2MeOH	**6**·$4H_2O$	**7**·$2MeOH\cdot 4H_2O$	**9**·$9.5H_2O$
Formula	$C_{40}H_{56}F_6N_6O_{12}Zn$	$C_{27}H_{33}ClCuF_3N_5O_7$	$C_{29}H_{40}ClCuF_3N_6O_8$	$C_{44}H_{61}F_6MnN_8O_{15.5}$
Crystal system	Triclinic	Triclinic	Monoclinic	Triclinic
Space group	*P*–1	*P*–1	P 2_1/n	*P*–1
a (Å)	8.9476(6)	10.2567 (3)	13.8736(5)	13.1414(4)
b (Å)	10.4849(6)	10.6402(5)	15.2501(6)	13.2581(5)
c (Å)	12.5638(8)	15.2662(7)	17.0142(7)	15.2976(5)
α (◦)	97.450(5)	91.425(4)	90	88.007(3)
β (◦)	101.316(5)	105.397(4)	110.409(4)	84.682(2)
γ (◦)	98.746(5)	109.119(4)	90	70.902(3)
Volume ($Å^3$)	1126.74(13)	1504.71	3373.79	2507.73
Z	1	2	4	1
T (K)	150	150	150	150
D_{calc}	1.462	1.535	1.490	1.480
Reflections	10485	6911	7730	11463
Reflections with I>2σ(I)	4255	4582	6418	9082
R (I>2σ(I))	0.0573	0.0828	0.0348	0.0526
wR (I>2σ(I))	0.1556	0.2213	0.0832	0.1331
Gof	1.046	1.036	1.054	1.029

FIGURES

(A)

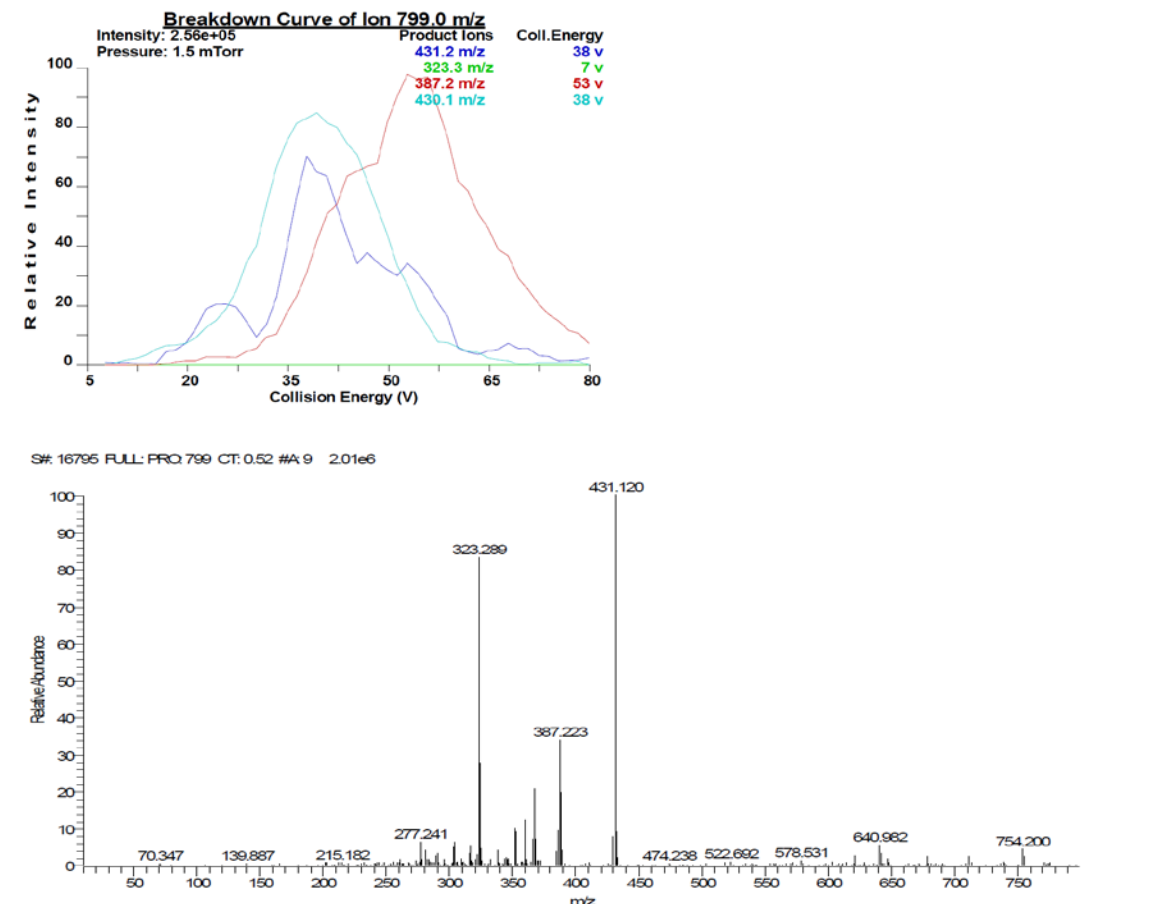

(B)

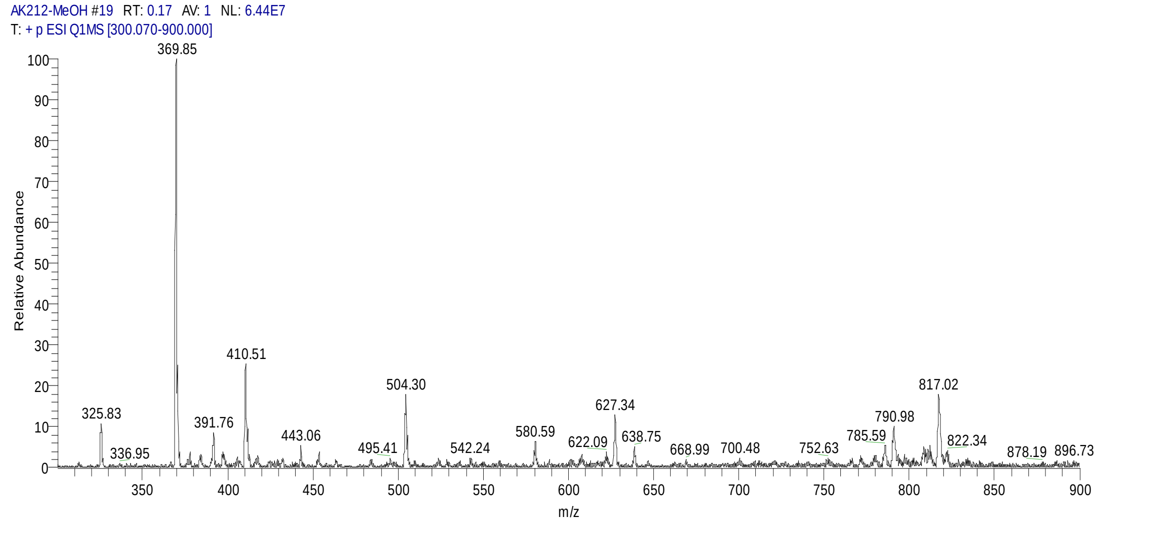

Figure S1. (A) ESI–MS(+) of complex **1** (m/z = 799) in MeOH. (B) ESI–MS(+) of complex **2** (m/z = 878.19) in MeOH.

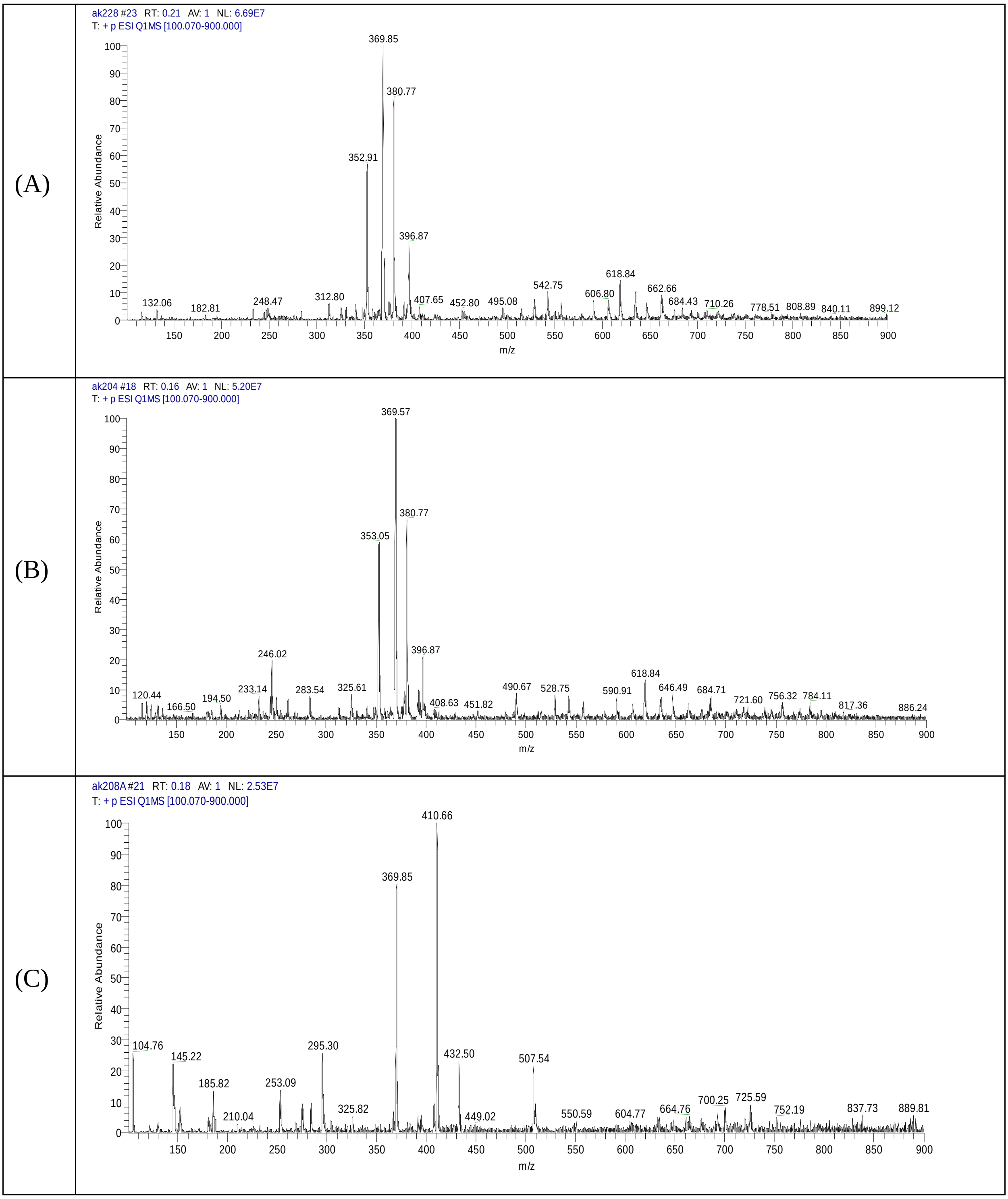

Figure S2. (A) ESI–MS(+) of complex **3** (m/z = 899.12) in MeOH. (B) ESI–MS(+) of complex **4** (m/z = 886.24) in MeOH. (C) ESI–MS(+) of complex **5** (m/z = 899.81) in MeOH.

(A)

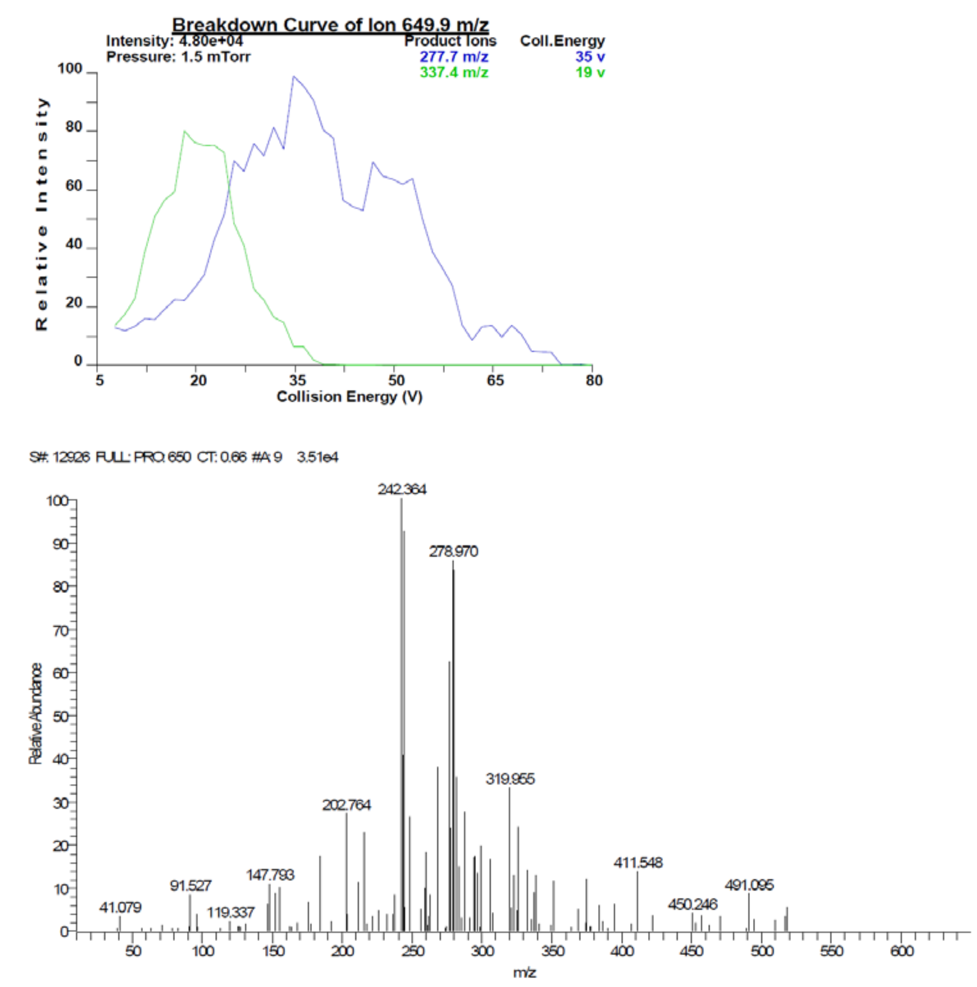

(B)

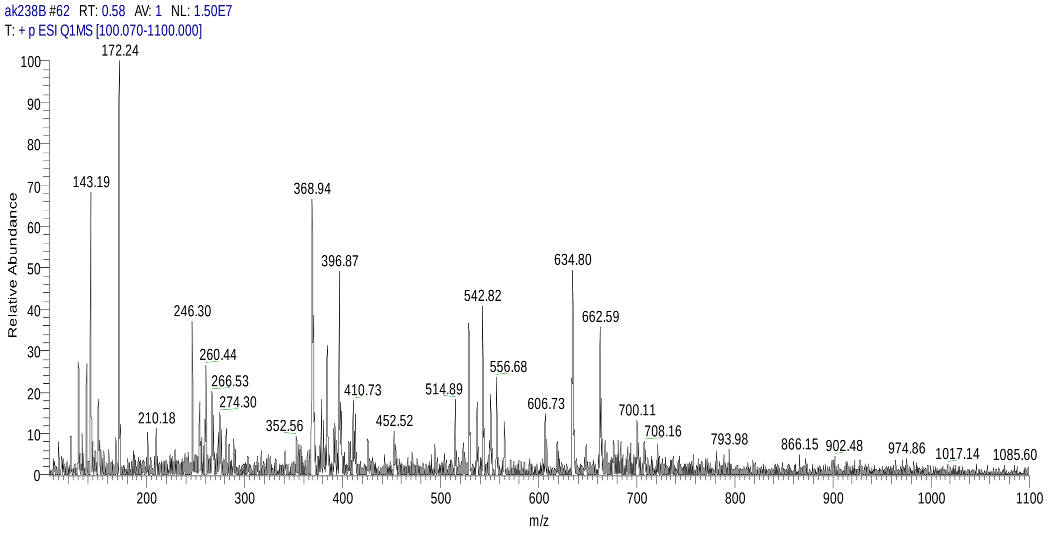

Figure S3. (A) ESI–MS(+) of complex **8** (m/z = 649.9) in MeOH. (B) ESI–MS(+) of complex **9** (m/z = 974.86) in MeOH.

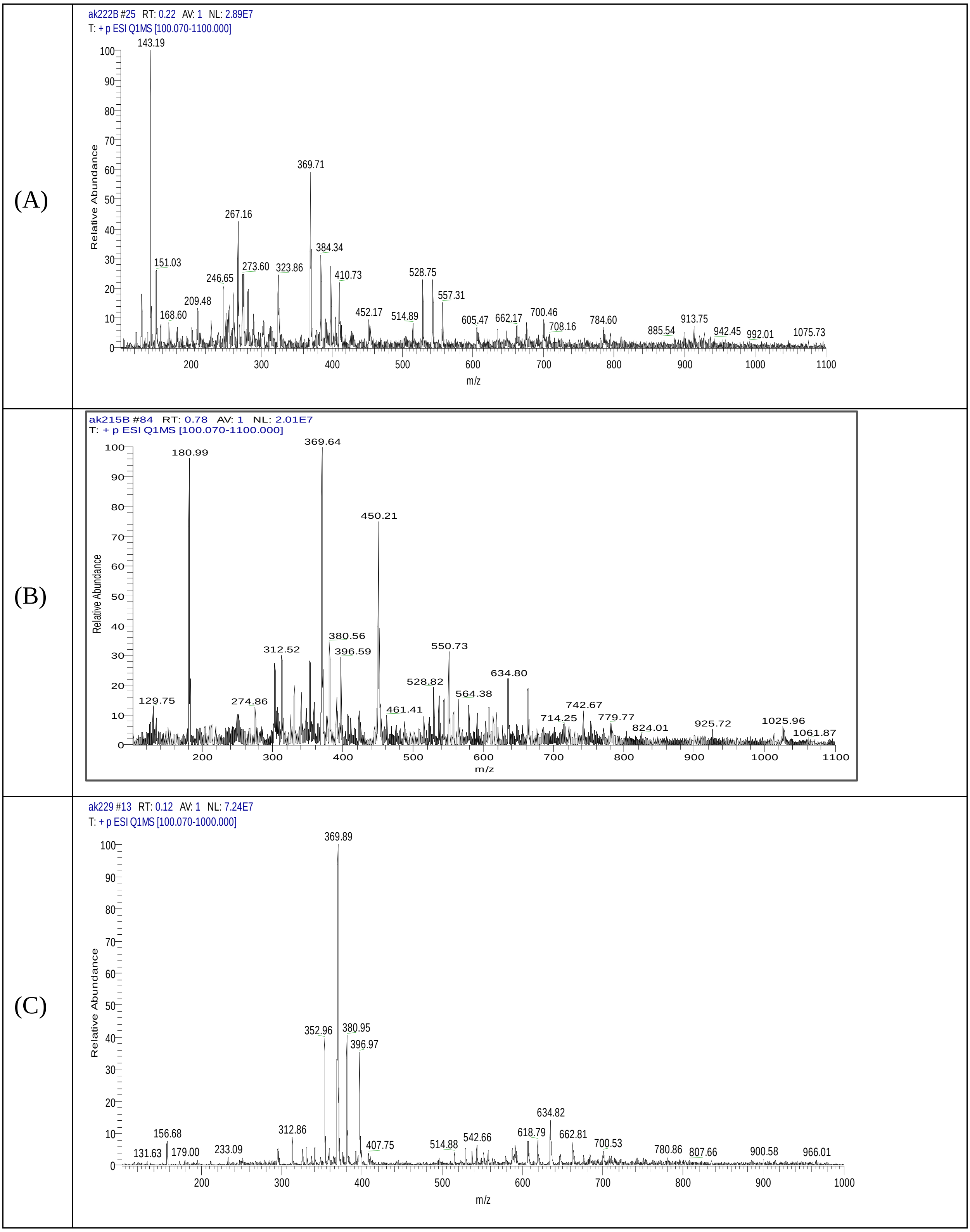

Figure S4. (A) ESI–MS(+) of complex **10** (m/z = 992.01) in MeOH. (B) ESI–MS(+) of complex **11** (m/z = 1025.96) in MeOH. (C) ESI–MS(+) of complex **12** (m/z = 966.01) in MeOH.

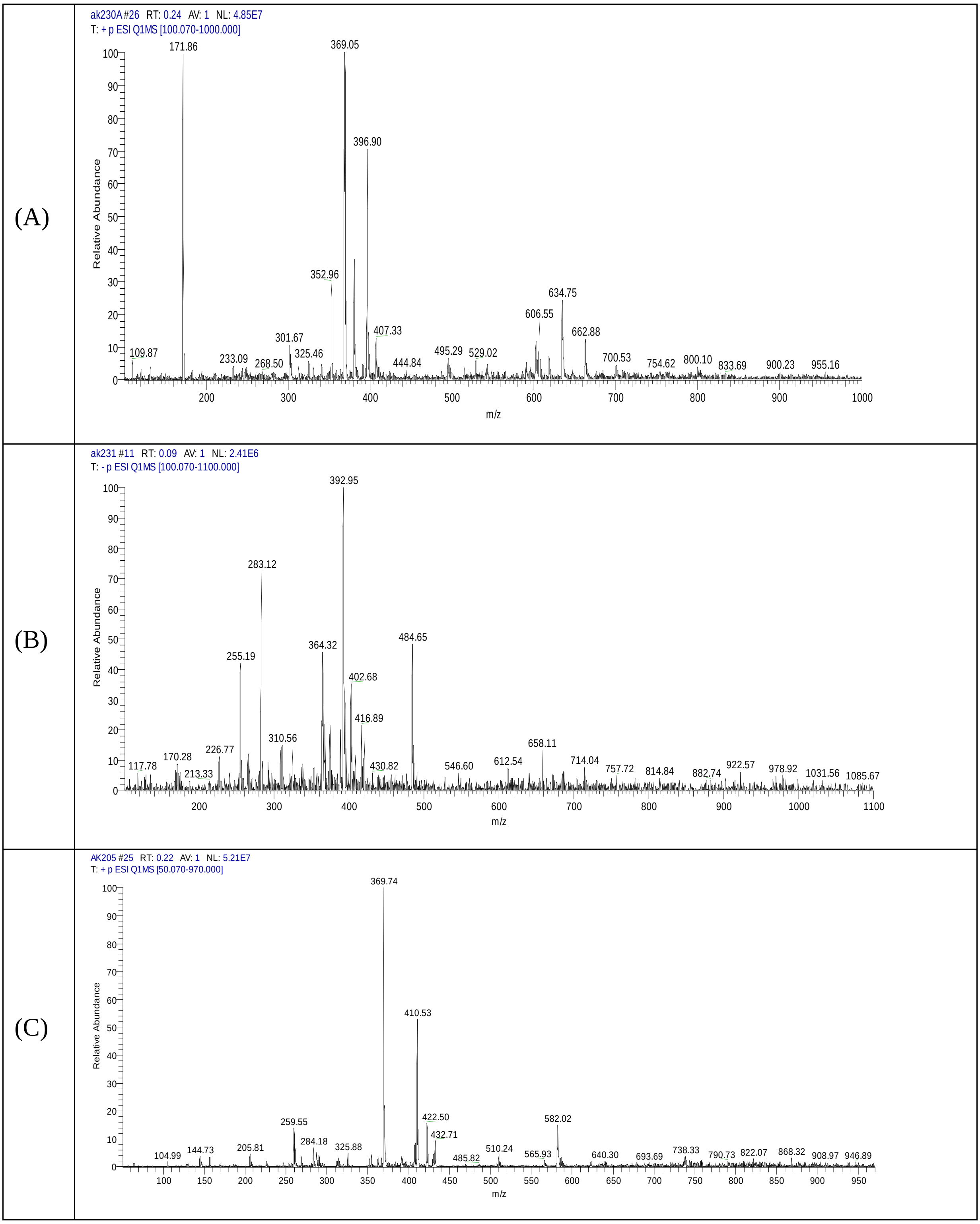

Figure S5. (A) ESI–MS(+) of complex **13** (m/z = 955.16) in MeOH. (B) ESI–MS(–) of complex **14** (m/z = 978.92) in MeOH. (C) ESI–MS(+) of complex **15** (m/z = 946.89) in MeOH.

<table>
<tr><td>(A)</td><td>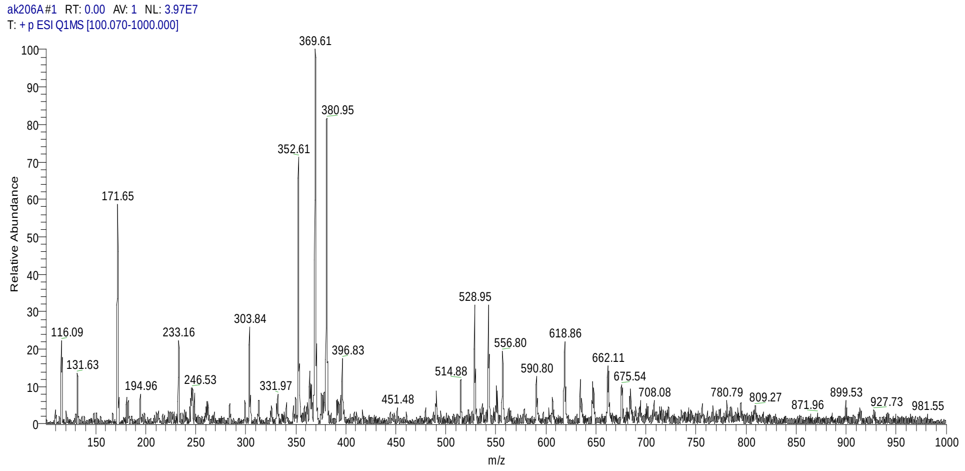
</td></tr>
<tr><td>(B)</td><td>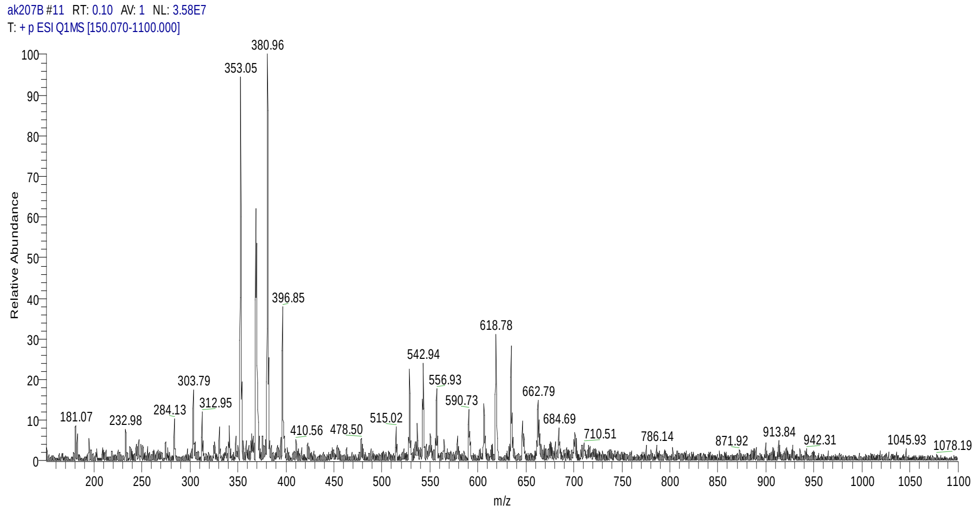
</td></tr>
<tr><td>(C)</td><td></td></tr>
</table>

Figure S6. (A) ESI–MS(+) of complex **16** (m/z = 981.55) in MeOH. (B) ESI–MS(+) of complex **17** (m/z = 1045.93) in MeOH.

(A)

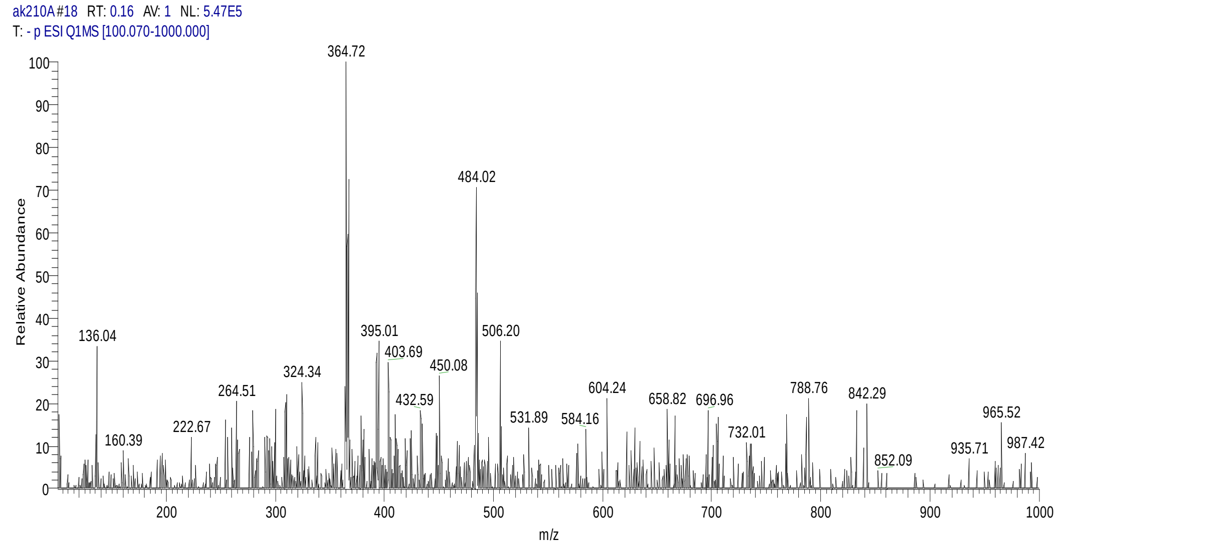

(B)

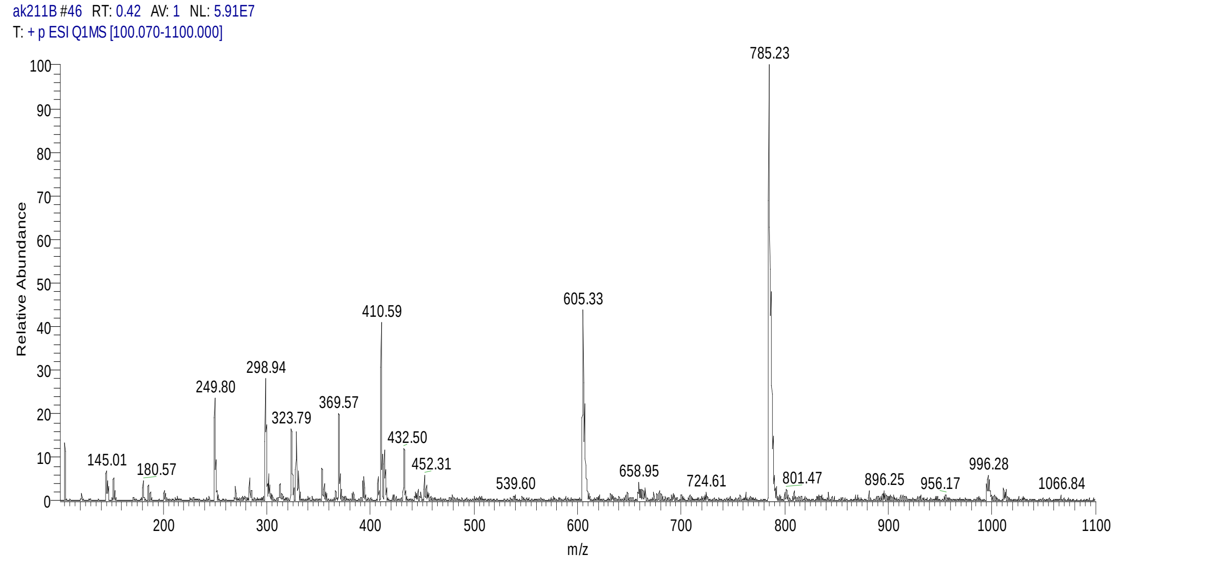

Figure S7. (A) ESI–MS(–) of complex **18** (m/z = 965.52) in MeOH. (B) ESI–MS(+) of complex **19** (m/z = 966.28) in MeOH.

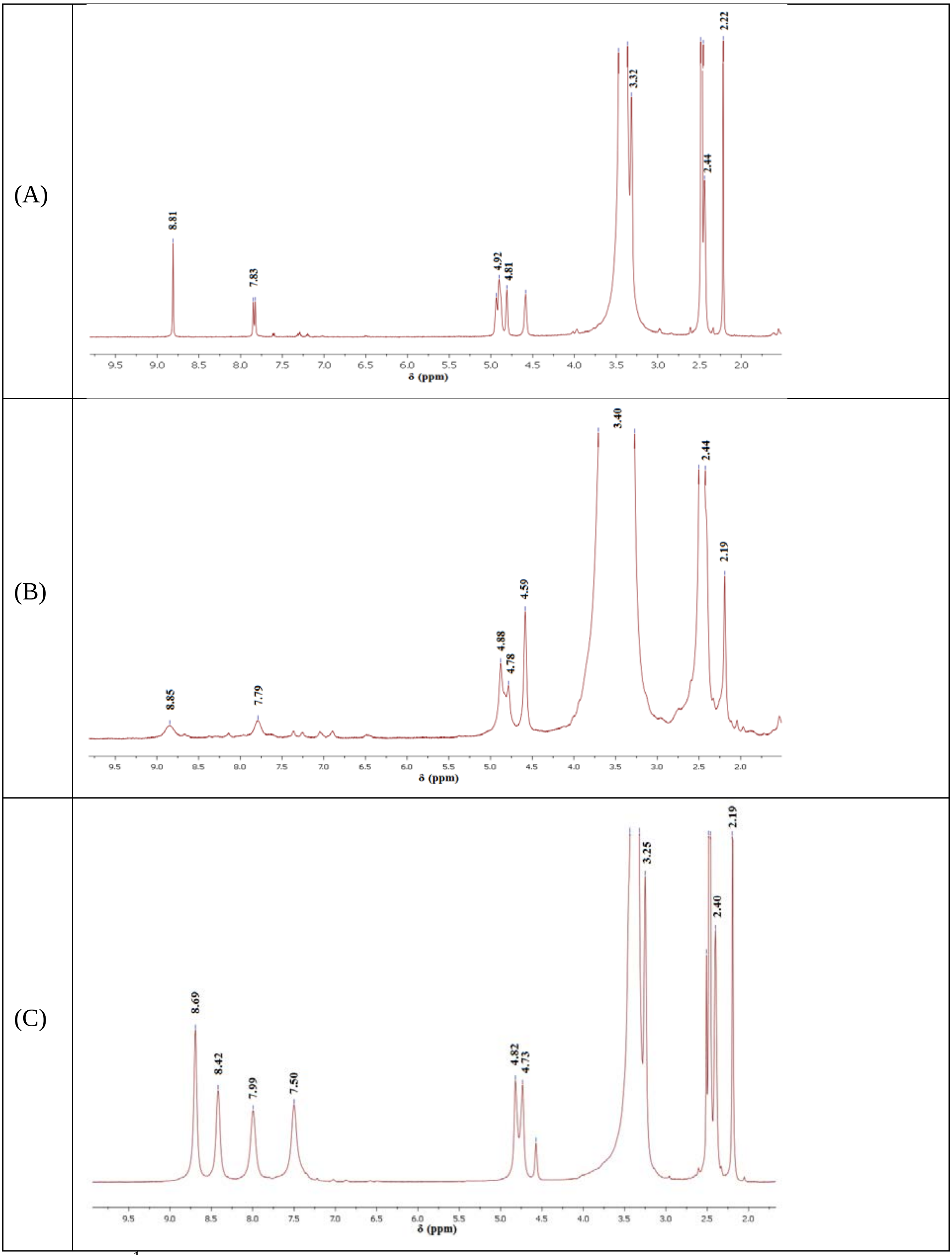

Figure S8. ^{1}H NMR spectrum of (A) Hflrx in DMSO–d_6, (B) [Zn(flrx)$_2$(MeOH)$_2$] in DMSO–d_6, and (C) [Zn(flrx)$_2$(bipy)] in DMSO–d_6.

(A)

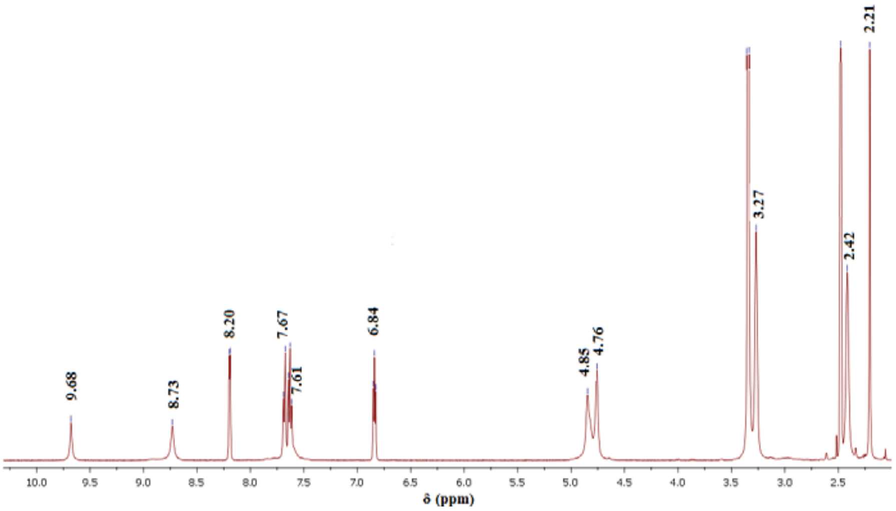

(B)

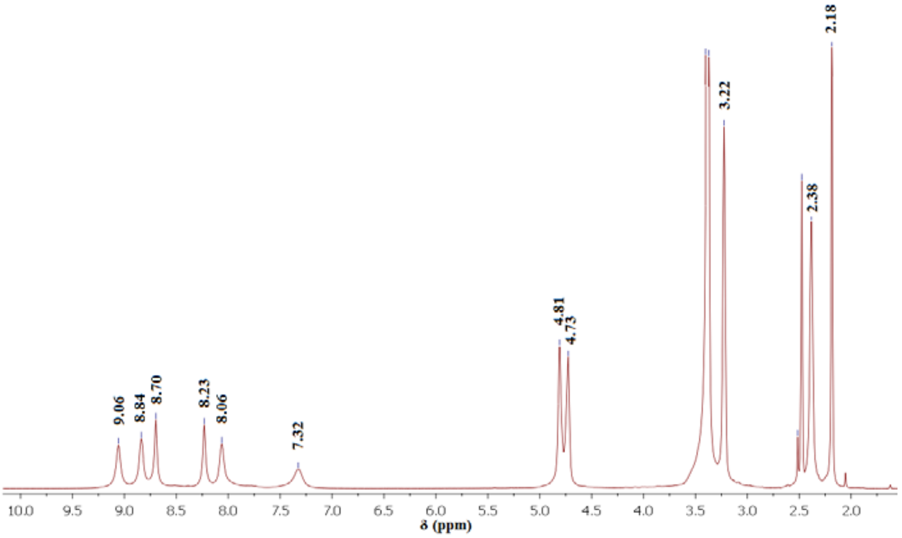

Figure S9. ^{1}H NMR spectrum of (A) [Zn(flrx)$_2$(bipyam)] in DMSO–d$_6$ and (B) [Zn(flrx)$_2$(phen)] in DMSO–d$_6$.

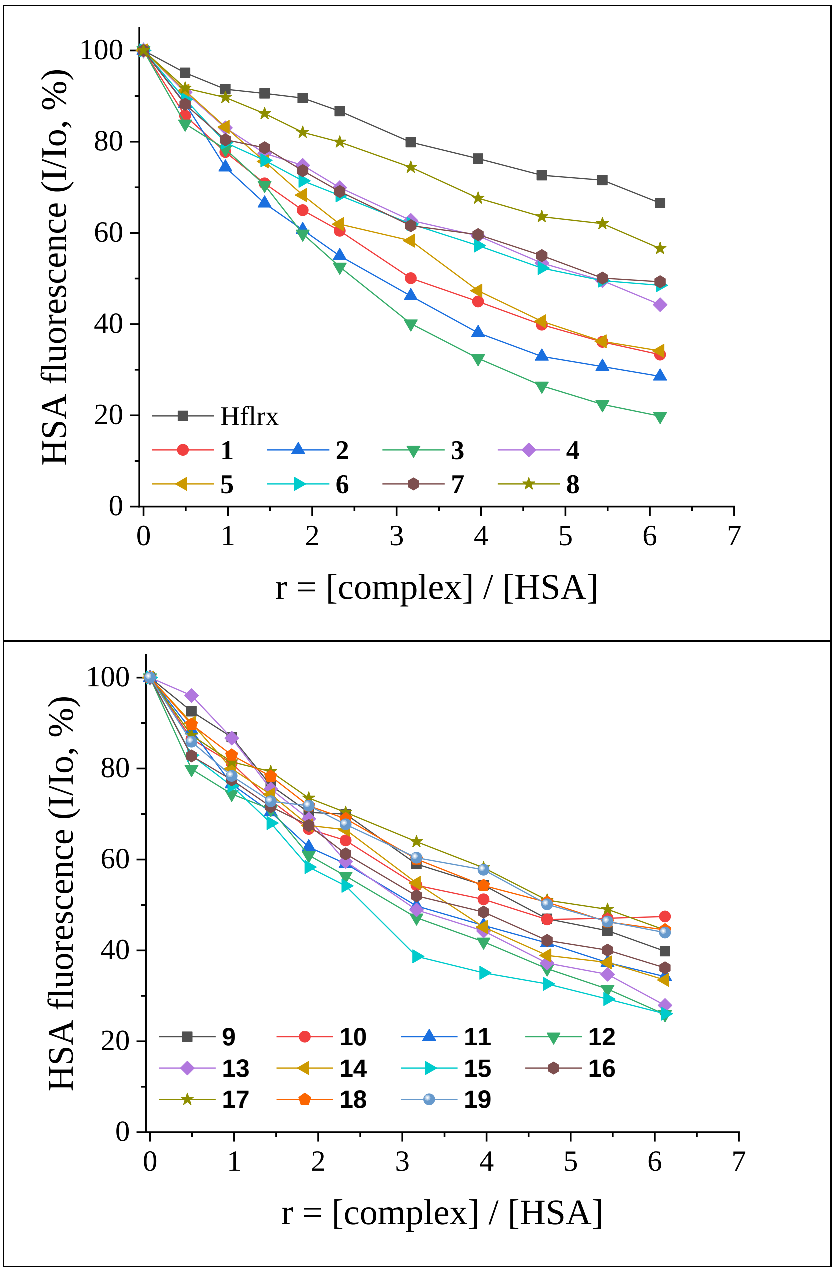

Figure S10. Plot of % relative intensity HSA–fluorescence emission band at $\lambda_{em,max}$ = 351 nm (I/I_o, %) *versus* r (r = [complex]/[HSA]) in the presence of Hflrx and its complexes **1–19** (66.5% of the initial fluorescence intensity for Hflrx, 33.3 % for **1**, 28.5% for **2**, 19.8% for **3**, 44.3% for **4**, 34.1% for **5**, 48.5% for **6**, 49.3% for **7**, 56.5% for **8**, 39.8% for **9,** 47.4% for **10,** 34.2 % for **11**, 25.9% for **12**, 27.9% for **13**, 33.5% for **14**, 26.1% for **15**, 36.1% for **16**, 44.5% for **17**, 44.5% for **18**, and 43.9% for **19**).

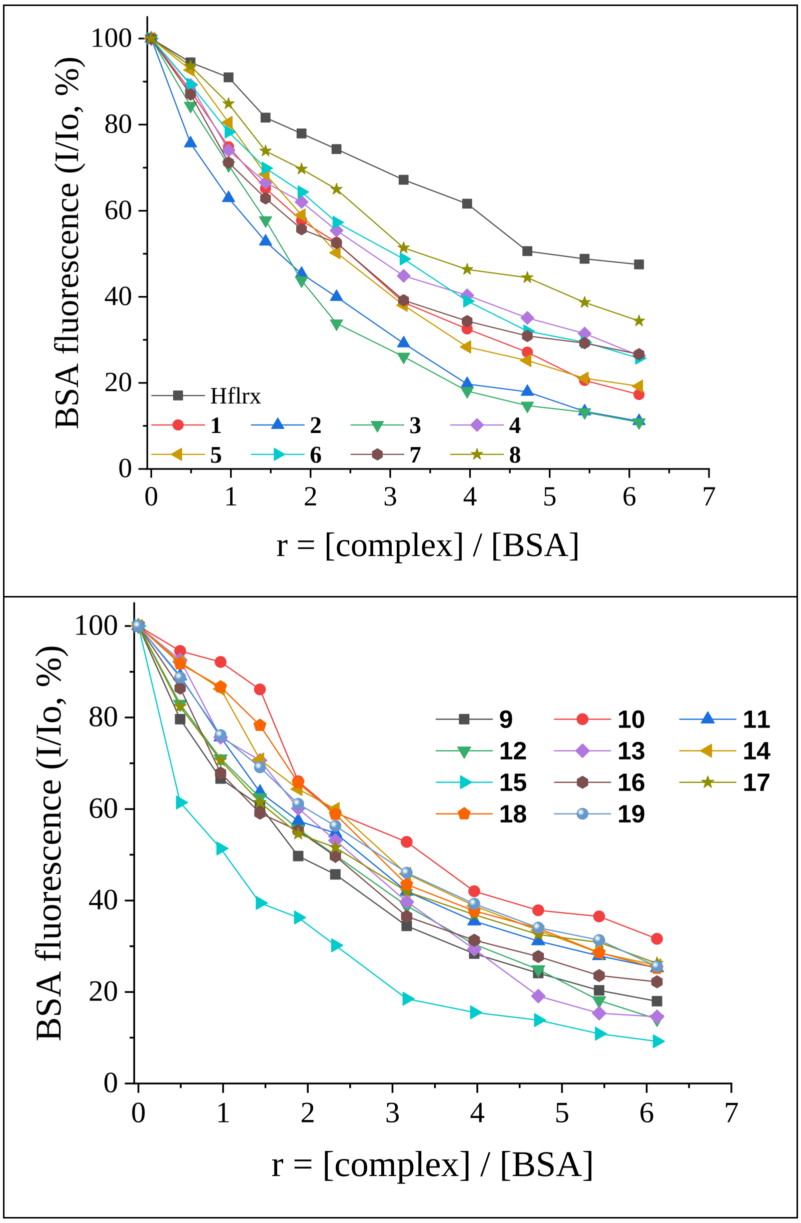

Figure S11. Plot of % relative intensity BSA–fluorescence emission band at $\lambda_{em,max}$ = 342 nm (I/I_o, %) *versus* r (r = [complex]/[BSA]) in the presence of Hflrx and its complexes **1–19** (47.5% of the initial fluorescence intensity for Hflrx, 17.3 % for **1**, 11.1% for **2**, 10.8% for **3**, 26.3% for **4**, 19.2% for **5**, 25.7% for **6**, 26.6% for **7**, 34.4% for **8**, 18.0% for **9,** 31.6% for **10,** 25.2 % for **11**, 14.1% for **12**, 14.6% for **13**, 25.8% for **14**, 9.2% for **15**, 22.2% for **16**, 26.3% for **17**, 25.2% for **18**, and 25.6% for **19**).

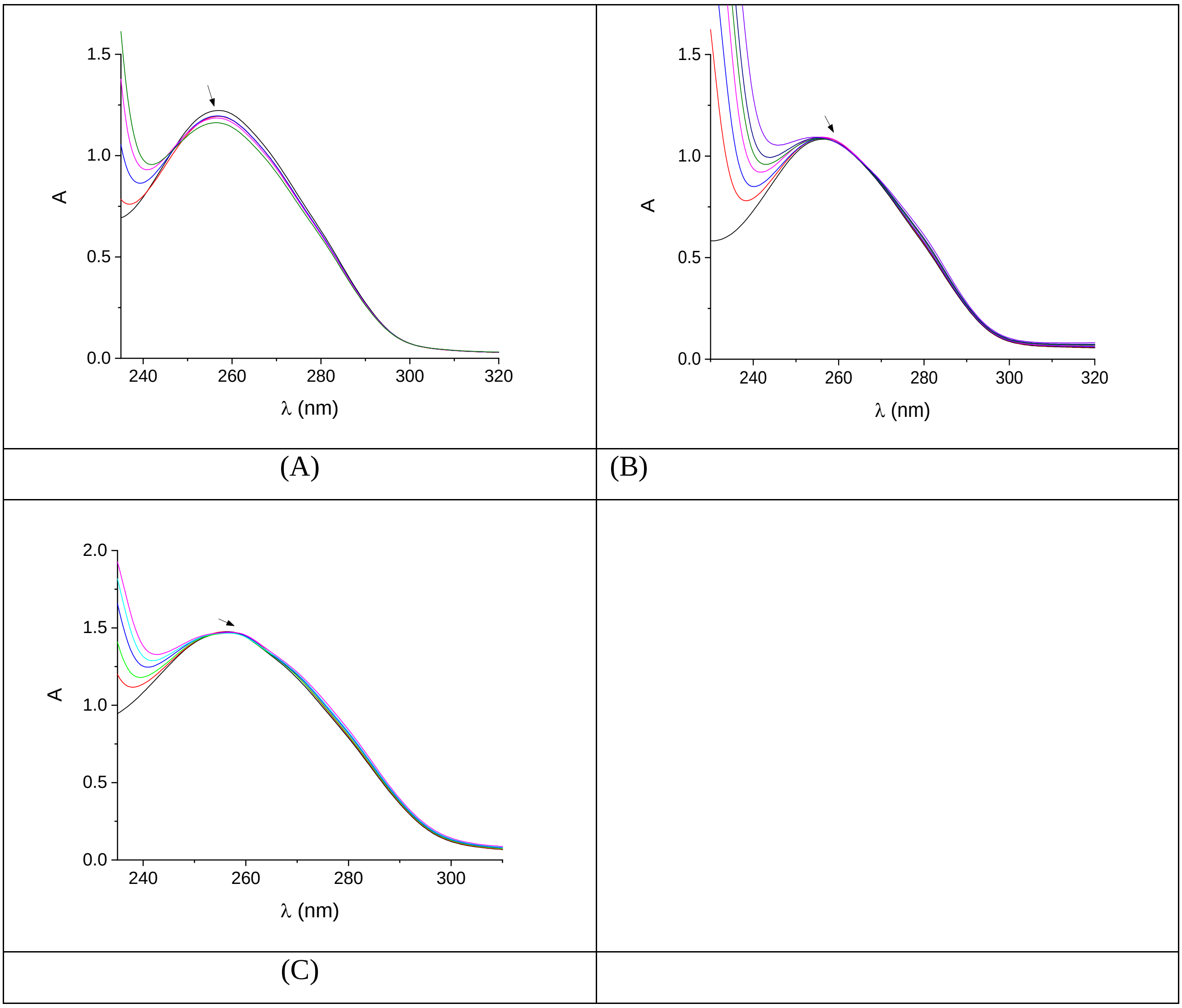

Figure S12. UV–vis spectra of a buffer solution (150 mM NaCl and 15 mM trisodium citrate at pH 7.0) containing CT DNA upon addition of increasing amounts of (A) Hflrx, (B) complex **5**, and (C) complex **11**. The arrows show the changes upon addition of increasing amounts of the compound.

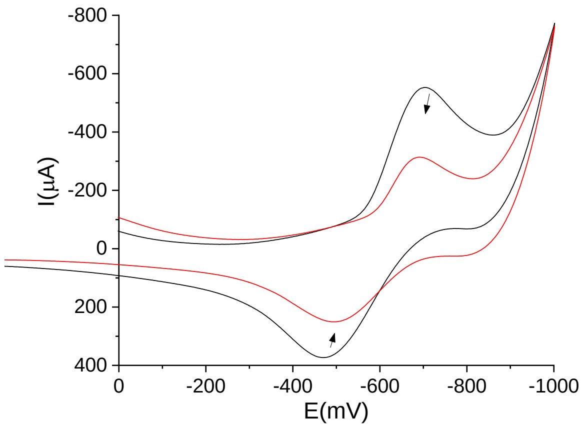

(A)

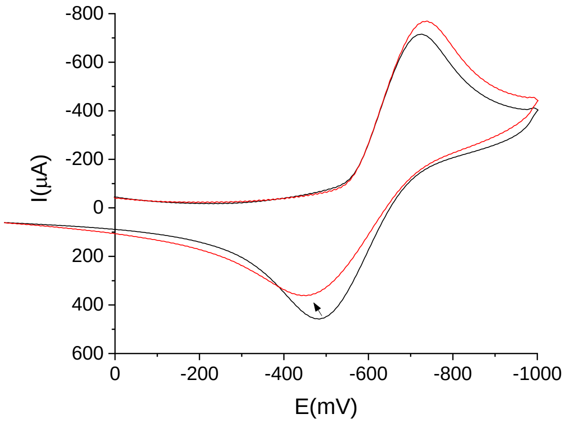

(B)

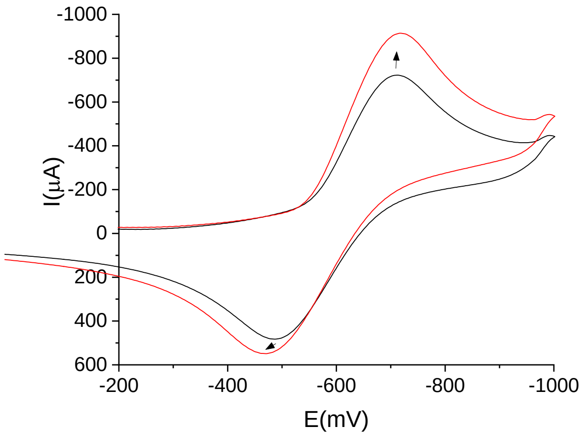

(C)

Figure S13. Cyclic voltammogram of 0.4 mM 1/2 DMSO/buffer (containing 150 mM NaCl and 15 mM trisodium citrate at pH = 7.0) solution of complex (A) **2**, (B) **4** and (C) **19,** in the absence (black line) or presence (red line) of CT DNA. Scan rate = 100 mV s^{-1}. Supporting electrolyte = buffer solution. The arrows show the changes upon addition of CT DNA.

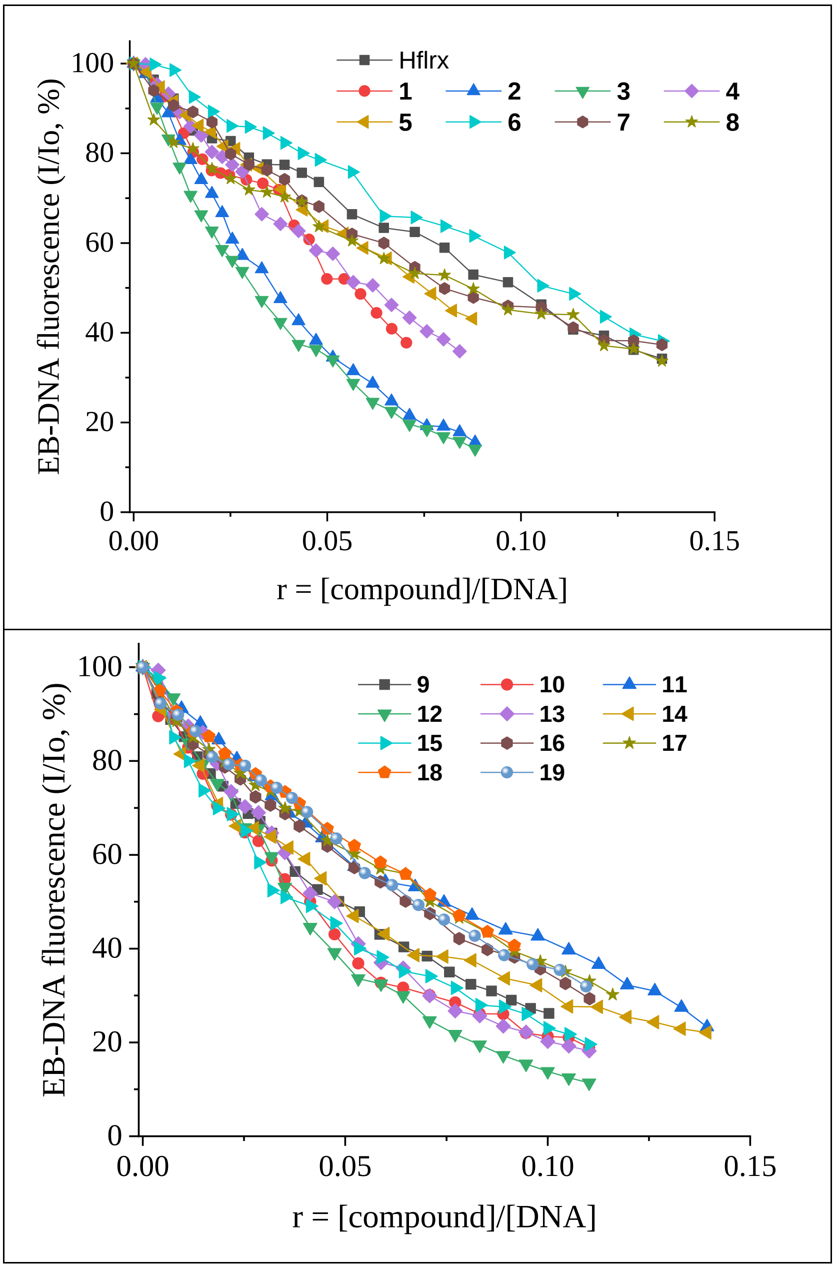

Figure S14. Plots of EB–DNA relative fluorescence emission intensity at $\lambda_{emission}$ = 592 nm (%) versus *r* (*r* = [complex]/[DNA]) in the presence of Hflrx and complexes **1–19** (up to 34.2 % of the initial EB–DNA fluorescence emission intensity for Hflrx, 37.8 % **1,** 15.6 % for **2**, 14.1 % for **3**, 35.9 % for **4**, 43.1 % for **5,** 38.1 % for **6**, 37.3 % for **7**, 33.6 % for **8,** 26.2 % for **9,** 18.9% for **10,** 23.3 % **11,** 11.4 % for 1**2**, 18.2 % for **13**, 22.1 % for **14**, 19.6 % for **15,** 29.3 % for **16**, 30.2 % for **17**, 40.6 % for **18** and 31.9 % for **19**).
